# Developmental exposure to a human-relevant PCB mixture: impacts on PCB congeners, metabolites, and drug-metabolizing enzymes in the bladder of post-weaning mice

**DOI:** 10.1007/s00204-026-04498-6

**Published:** 2026-07-04

**Authors:** Hui Wang, Alex Goskowicz, Audrey Spiegelhoff, Conner L. Kennedy, Monica M. Ridlon, Rachel F. Marek, Hans-Joachim Lehmler, Kimberly P. Keil Stietz

**Affiliations:** 1 Department of Occupational and Environmental Health, The University of Iowa, Iowa City, IA 52246 USA; 2 Department of Comparative Biosciences, University of Wisconsin-Madison, 2015 Linden Dr. Madison, Madison, WI 53706 USA; 3 Department of Civil & Environmental Engineering, The University of Iowa, Iowa City, IA 52246 USA

**Keywords:** Bladder function, Cytochrome P450, Lower urinary tract symptoms, OH-PCB, Phase I metabolism, Pregnancy

## Abstract

Many women suffer from symptoms associated with dysfunction of the lower urinary tract, including incontinence and overactive bladder, especially during pregnancy. While causes are likely multifactorial, environmental factors, such as exposure to polychlorinated biphenyls (PCBs), have been shown to alter voiding in adult female mice. However, the distribution of PCBs and their metabolites to the bladder and urine, as well as their effects on bladder drug-metabolizing enzyme expression after dosing with PCB mixtures, remains largely unexplored. Female C57BL/6J mice were exposed to MARBLES PCB mixture (0, 0.1, 1, and 6 mg/kg/d) through gestation and lactation, and post-weaning bladder, urine, blood, adipose, and liver were collected for measurement of PCBs and OH-PCBs, and for abundance of cytochrome P450 (*Cyp*) transcripts in liver and bladder. PCBs and OH-PCBs were detected in all tissues in a dose-dependent manner. Bladder, adipose, and liver retained parent PCBs while OH-PCBs were predominant in urine and blood. *Cyp1a2* mRNA abundance was elevated in the liver of the high dose PCB group versus the vehicle, and the same trend was observed in the bladder. These results define the signatures of PCBs and OH-PCBs in the bladders and urine of post-weaning dams exposed to the MARBLES mixture of PCBs and provide a reference for comparison with accumulation in more commonly studied tissues. Our results also provide new insights into the regulation of *Cyp1a2* in the liver and bladder, changes that may play a role in how environmental exposures in adulthood alter voiding function.

**Supplementary Information:**

The online version contains supplementary material available at https://doi.org/10.1007/s00204-026-04498-6.

## Introduction

Lower urinary tract symptoms (LUTS) encompass a broad range of urinary dysfunctions, including storage, voiding, and post-voiding disturbances, which manifest as incontinence, overactive or underactive bladder, nocturia, a weak urinary stream, incomplete emptying, dysuria, and pelvic pressure. Globally, approximately 63% of adults experience some form of LUTS, and 31% report symptoms of moderate or severe intensity (Huang et al. 2023). The prevalence of moderate-to-severe LUTS has risen over recent decades, increasing from about 27% in the 1990s to 36% in the 2010s, highlighting its importance for public health. Women are particularly affected by LUTS, with 20.6% of women worldwide reporting moderate to severe symptoms (Huang et al. 2023).

Although often associated with aging, LUTS can also emerge during and after pregnancy, when physiological, hormonal, and environmental factors interact to compromise bladder function (Balik et al. 2016; Li et al. 2019; Kim et al. 2020). Pregnant women often experience urinary urgency, frequency, and incontinence, and these symptoms can sometimes be worsened by pre-existing or co-occurring conditions such as overweight, obesity, gestational diabetes, and preeclampsia (Liang et al. 2020; Yavuz et al. 2022). Animal studies also suggest that pregnancy might raise the risk of urinary dysfunction (Rodriguez et al. 2004; Mustafa 2018), and incontinence is also frequently seen in female dogs following spaying (Applegate et al. 2018; Pegram et al. 2019). While LUTS are often comorbid with other conditions or trauma, the factors that give rise to these symptoms remain poorly characterized; whether environmental factors, such as exposure to persistent organic pollutants (POPs), contribute to LUTS remains underexplored.

Polychlorinated biphenyls (PCBs) are among the most concerning of the POPs. Although banned in the late 1970s, PCBs persist in the environment, bioaccumulate through the food chain, and remain detectable in human serum, breast milk, and placenta (DeKoning and Karmaus 2000; Marek et al. 2013). In rodents, PCB exposure during gestation alters maternal physiology and affects maternal organ systems, including the bladder, liver, and endocrine tissues (Alarcon et al. 2021; Lavery et al. 2023). PCBs and their hydroxylated metabolites (OH-PCBs) may disrupt smooth muscle contractility and hormone signaling, thereby potentially influencing bladder function in female animal models or humans (Kitamura et al. 2005; Parker et al. 2018; Lavery et al. 2023). PCBs undergo extensive phase I metabolism and can increase their own metabolism to OH-PCBs by inducing hepatic CYP enzymes (Grimm et al. 2015). Earlier studies highlighted the presence of drug-metabolizing enzymes in extrahepatic organs, such as the bladder, which may contribute to local toxicity (Gundert-Remy et al. 2014). CYP-mediated phase 1 metabolism of environmental toxicants into carcinogenic active metabolites has been implicated in bladder cancer (Bonassi et al. 1989; Clavel et al. 1994; Landi et al. 1999; Kasperczyk et al. 2025). Additionally, PCB metabolism and excretion may be modified by physiological adaptations to gestation, including changes in renal clearance, hepatic enzyme expression, and gut absorption (Su et al. 2024).

Our previous work demonstrated that developmental exposure of mice to the MARBLES PCB mixture (MARBLES mix) altered voiding physiology and resulted in PCB accumulation in bladder tissue (Keil Stietz et al. 2021; Kennedy et al. 2022; Ridlon et al. 2025). This human-relevant, synthetic PCB mixture closely resembles the congener profile detected in serum from pregnant women in the Markers of Autism Risk in Babies – Learning Early Signs (MARBLES) study, who are at a higher risk of having another child with a neurodevelopmental disorder (Hertz-Picciotto et al. 2018). Despite increasing evidence linking PCB exposure to LUTS, it remains unclear whether PCBs and their metabolites accumulate in the bladder of post-weaning dams exposed to PCBs through gestation and lactation, and whether maternal PCB metabolism plays a role in urinary dysfunction. Additionally, the regulation of bladder-specific CYP enzymes after gestation and lactation in response to PCB exposure has not been studied in post-weaning female mice.

To address these knowledge gaps, we exposed female mice to the MARBLES mix via diet starting two weeks before mating and continuing through gestation and lactation. We measured PCB and OH-PCB levels in adipose tissue, bladder, blood, liver, and urine using gas chromatography-tandem mass spectrometry (GC-MS/MS) and assessed CYP expression in the bladder and liver with real-time quantitative PCR (RT-qPCR). We detected PCBs and OH-PCBs in the tissues of post-weaning female mice and observed that *Cyp1a2* levels increased following PCB exposure. These observations underscore the need to further investigate how developmental exposure to a human-relevant PCB mixture alters maternal PCB metabolism and bladder-specific responses.

## Materials and methods

### Chemicals and analytical standards

The nomenclature of PCBs is based on the table of PCB congeners published by the US EPA (EPA, 2023). The naming of OH-PCB congeners follows the nomenclature suggested by Maervoet and co-workers, where the first number shows the position of the OH group and the second number indicates the parent PCB (Maervoet et al. 2004). All chemicals and analytical standards are described in the Supplementary Information. The SMILES structures of all PCB congeners and MeO-PCB (methylated forms of OH-PCBs) are reported in Table S1 and S2, respectively.

The MARBLES mix was prepared to approximate the PCB congeners profile detected in pregnant women in the MARBLES cohort (Hertz-Picciotto et al. 2018). The composition of the MARBLES mix is as follows: PCB28 (48.2%), PCB11 (24.3%), PCB118 (4.9%), PCB101 (4.5%), PCB52 (4.5%), PCB153 (3.1%), PCB180 (2.8%), PCB149 (2.1%), PCB138 (1.7%), PCB84 (1.5%), PCB135 (1.3%), and PCB95 (1.2%) (Sethi et al. 2019). The PCB congeners used to prepare the MARBLE mis were synthesized and authenticated by the Synthesis Core of the University of Iowa Superfund Research Program (Li et al. 2018; Sethi et al. 2019).

### Animals and exposure

All animal procedures were conducted in accordance with the NIH Guide for the Care and Use of Laboratory Animals and were approved by the University of Wisconsin-Madison Animal Care and Use Committee (protocol #V006099). The study design and conduct of the animal study have been reported following the ARRIVE guidelines (Percie du Sert et al. 2020) in earlier publications (Kennedy et al. 2022; Lavery et al. 2023). Briefly, C57BL/6J mice (RRID: IMSR_JAX:000664, Jackson Labs, Bar Harbor, ME, USA) were housed in clear plastic cages with corn cob bedding. Feed (Diet #2019 for breeders and 2020x for maintenance; Teklad, Indianapolis, IN, USA) and water were available *ad libitum*. Housing was maintained on a 12 h light and dark cycle at 22 ± 2 °C.

Female nulliparous mice (*n* = 11–13), aged approximately 50 ± 8 days, were exposed to 0 (vehicle control), 0.1 (low dose), 1.0 (medium dose), or 6.0 mg/kg (high dose) MARBLES mix per day. These doses have been shown to produce biological effects in both dams and their offspring, without causing overt toxicity or adversely affecting reproductive outcomes (Kennedy et al. 2022; Lavery et al. 2023). Body mass was also not altered following exposure to the MARBLES mix (Table S3). For PCB administration, a 20 mg/mL PCB mix stock in organic peanut oil (Spectrum Organic Products, LLC, Melville, NY) was diluted in peanut butter (Trader Joe’s, Monrovia, CA) to make working stocks of 0.025, 0.25, and 1.5 mg PCB/g peanut butter to give to mice for oral daily consumption. Mice were dosed for two weeks, were then paired with a male, and dosing continued through mating, gestation, and lactation until collection (67 ± 10 days dosed) (Sethi et al. 2019; Keil Stietz et al. 2021).

To refine and reduce the number of animals, the tissues used here were generated as part of a larger study aimed at producing offspring (Kennedy et al. 2022). Additionally, to add relevance to voiding function, some dams underwent voiding assays (void spot assay, uroflow, and anesthetized cystometry) before being euthanized (Lavery et al. 2023). Bladder and liver were immediately collected following euthanasia, weighed, snap frozen, and stored at -80 °C. Blood was collected via cardiac puncture and placed in glass vials containing 80 µL of 7.5% EDTA, then stored at -80 °C. Urine was collected as free catch urine upon scuffing animals one day prior to voiding testing/collection, or was collected from a plexiglass plate after mice freely urinated through a grid during uroflow testing (Lavery et al. 2023). Urine was stored in glass vials at -80 °C. For the bladder, samples from 2 to 3 animals were pooled (*n* = 2–4 pools per exposure group) to generate enough tissue for PCB and metabolite quantification. Some of the pooled samples contain 1 tissue from a dam who failed to take care of their litter to weaning, but continued to be dosed to the wean date. All other tissues were not pooled, with *n* = 5–6 mice per experimental group.

### PCB and OH-PCB extractions from tissues

PCB and OH-PCB were extracted from blood, tissues, or urine using a liquid-liquid extraction method, as reported previously (Wang et al. 2026), and detailed in the Supplementary Information. Briefly, pre-weighted adipose tissue (143 ± 18 mg, *n* = 5–6), liver (28 ± 4 mg, *n* = 5–6), or pooled bladder samples (45 ± 8 mg, *n* = 2–4 pools) were homogenized in 3 mL of 2-propanol. Surrogate standards (10 ng) were added to each sample before extraction. PCBs and OH-PCBs were extracted using a mixture of diethyl ether and hexane, the organic layer was washed with 5 mL of 0.1 M phosphoric acid, and OH-PCBs were derivatized to methoxylated PCB derivatives using diazomethane. Further cleanup was performed by passing the extracts through a sulfuric acid–silica gel cartridge (1:2, w/w). The final extracts were spiked with an internal standard (10 ng) and quantified by gas chromatography–tandem mass spectrometry (GC–MS/MS).

Blood samples (415 ± 15 mg, *n* = 5–6) were processed using the same extraction protocol with slight modifications. Due to the high abundance of PCB conjugates in urine, urine samples (282 ± 146 mg, *n* = 5–6) were first subjected to enzymatic deconjugation with sulfatase (type H-2 from Helix pomatia; Sigma-Aldrich, St. Louis, MO, USA) to transform PCB conjugates into OH-PCBs. Following deconjugation, urine samples were extracted and processed using the same procedure as described for blood samples; see the Supplementary Information.

### GC-MS/MS instrumental analysis

The quantification of PCB and OH-PCB (in the form of methoxylated PCBs) was performed using a GC system (Agilent 7890B) coupled with a triple quadrupole mass spectrometer (Agilent 7000D). The GC was equipped with an SPB-Octyl capillary column with a poly (50% n-octyl/50% methyl siloxane) stationary phase (30 m length, 0.25 mm inner diameter, 0.25 μm film thickness; Supelco, Bellefonte, PA, USA). Instrumental parameters, specific precursor and product ions, and collision energies are described in the Supplementary Information. Specific precursor and product ions, and collision energies for each analyte were listed in Supplementary Table S4.

### Quality assurance/quality control (QA/QC) for PCB analysis

Method blanks, tissues from vehicle-only exposed animals, and ongoing precision and recovery (OPR) standards were extracted in parallel to the samples with each sample batch to ensure the rigor and reproducibility of the analyses. Analyte masses were corrected based on the recoveries of their corresponding surrogate standards (SS). PCB15 and PCB117 were used as SS for PCBs, while 4′-OH-PCB9, 4-OH-PCB91, and 4′-OH-PCB159 served as SS for OH-PCBs. Deuterium-labeled d-PCB30 (CDN Isotopes, Quebec, Canada) and PCB204 were used as internal standards (volume correctors). Method detection limits (MDLs) and limits of detection (LODs) were calculated using the formulas provided in the Supplementary Information. All QA/QC results, including SS recoveries, OPR recoveries, MDLs, and LODs, are summarized in Supplementary Tables S5–S7.

### RT-qPCR in the bladder and liver

The Cytiva RNAspin isolation kit was used according to the manufacturer’s instructions (Fisher Scientific, Waltham, MA, USA) to isolate RNA from frozen bladder and liver tissue. Briefly, tissues were homogenized in lysis buffer containing 10 µL of molecular grinding resin (G Biosciences, St Louis, MO, USA). cDNA was generated from 350 ng of RNA using GoScript Reverse Transcription Kit according to the manufacturer’s instructions (Promega, Madison, WI, USA). Real-time PCR was performed in 12 µL reactions consisting of 6 µL of SsoFast EvaGreen Supermix (Bio-Rad, Hercules, CA, USA), 0.5-1 µL of a combined 10 µM stock of forward and reverse primers, 3-3.5 µL of water, and 2 µL of cDNA. Primer3 or NCBI Primer Design and Primer BLAST were used to design and confirm primer targets. Primers were ordered from IDT (Coralville, IA, USA).

Primer efficiency was assessed through serial dilutions of cDNA to determine the optimal annealing temperature. Efficiencies of 90 to 111% were obtained for all primers used. Table S8 lists primer sequence and annealing temperatures. Samples were run in triplicate on a CFX Maestro real-time system with Bio-Rad CFX software. The PCR protocol consisted of 95 °C for 2 min, then 39 cycles of 95 °C for 5 s, annealing temperature specified in Table S8 for 30 s, followed by a final 95 °C for 5 s, 65 °C for 5 s, and ramp to 95 °C for 5 s to generate a melt curve. Relative mRNA abundance was determined using the delta CT method as described (Livak and Schmittgen 2001), and normalized to *Ppia* (bladder) or *Pgk1* (liver) abundance. Some low-abundance transcripts did not amplify in all samples, or outliers were detected as described in the statistics section. In the final analysis, a total of *n* = 3–5 mice per exposure group were used.

### Statistical analysis

Statistics were performed using GraphPad Prism (version 10.0.3, RRID: SCR_002798). PCB levels were normalized by tissue wet weight. To compare the PCB and OH-PCB profiles of two groups, the similarity coefficient cos θ (ranging from 0 to 1) was calculated using this formula:

$$\:\mathrm{cos}{\uptheta\:}=\frac{{\sum\:}_{i=1}^{n}\left({A}_{i}{B}_{i}\right)}{\:\sqrt{{\sum\:}_{i=1}^{n}\left({{A}_{i}}^{2}\right)}\sqrt{{\sum\:}_{i=1}^{n}\left({{B}_{i}}^{2}\right)}}$$

where A_i_  and B_i_ are the i^th^ components of dataset A and B, respectively. A cos θ value of 1 indicates identical profiles, whereas a value of 0 denotes complete dissimilarity (Davis 2002). For qPCR, data were assessed for normality using the Shapiro-Wilk and Kolmogorov-Smirnov tests. If the data passed normality tests, a one-way ANOVA with Tukey’s post hoc test was performed; if the data failed to pass normality tests, a Kruskal-Wallis test with Dunn’s multiple comparisons test was used. If the data passed the normality test but did not pass Bartlett’s test for assumptions of equal standard deviation, Welch’s ANOVA was performed. Prism’s ROUT method was used to identify and remove outliers. P values < 0.05 were considered significant and are indicated by an asterisk in the figures.

## Results and discussion

### PCB and OH-PCB levels in the bladder

**Fig. 1:**
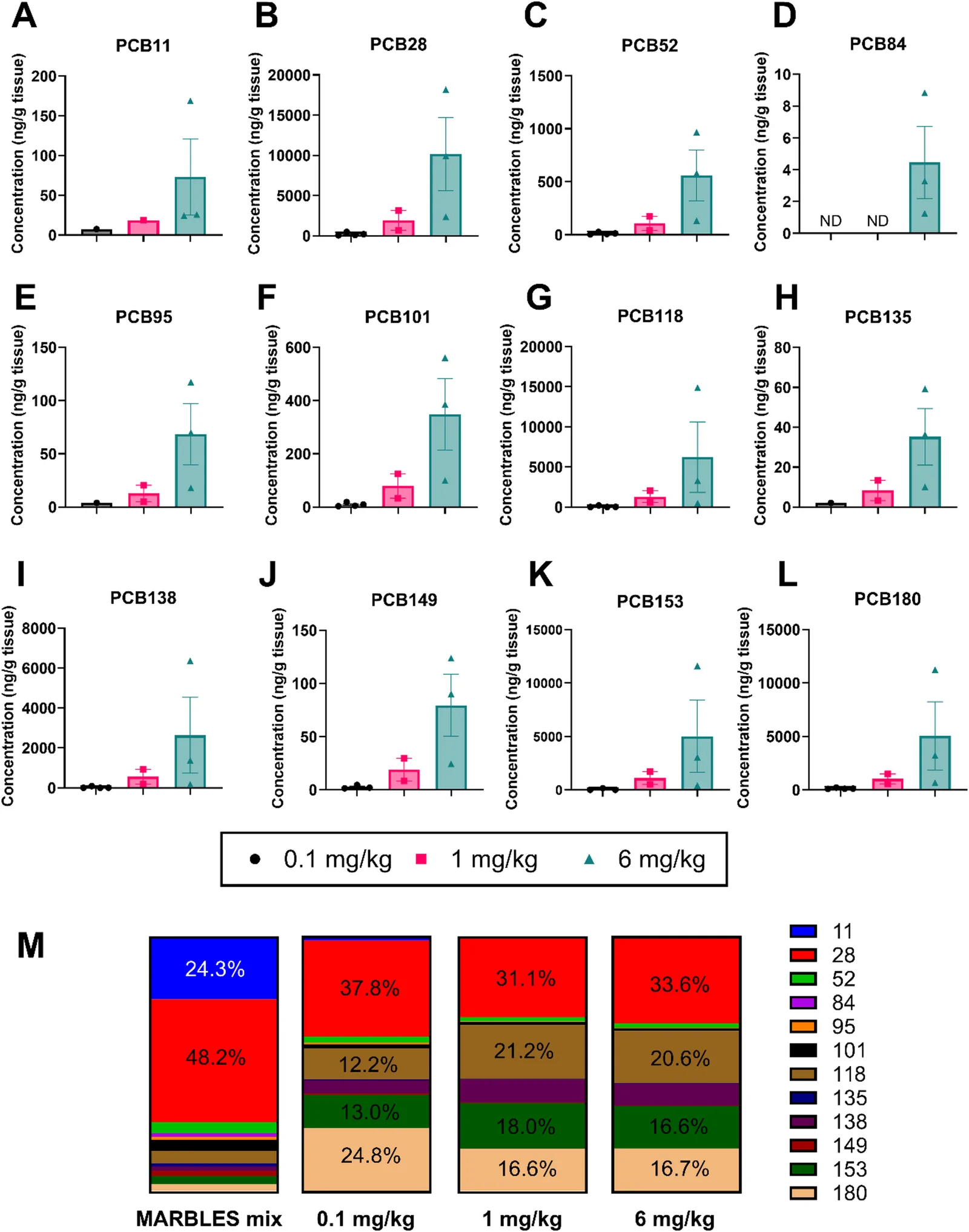
The concentration (ng/g ww) (**A**–**L**) and profile (%) (**M**) of 12 PCB congeners detected in the bladder of post-weaning dams. Due to their small size, bladders were pooled to form pools of approximately 50 mg; *n* = 2–4 pools per exposure group. Each bar was expressed as mean ± SEM, with individual values shown

All 12 PCB congeners were detected in the bladder, with levels increasing in a dose-dependent manner. PCB levels decreased in the order: PCB28 > PCB118 > PCB180 ≈ PCB153 > PCB138 > PCB52 > PCB101 > PCB149 > PCB11 > PCB95 > PCB135 > PCB84 (Fig. 1, Table S9). In the bladder, PCB28 was the most abundant PCB by weight, representing about one-third of the total PCB levels (Fig. 1M). Several higher-chlorinated PCBs, including PCB118, PCB153, and PCB180, were also retained in the bladder, ranging from 12% to 25% across dosing groups (Fig. 1M). In contrast, the proportion of PCB11 in the bladder was < 2%, compared to 24% in the MARBLES mix, likely due to its rapid metabolism (Hu et al. 2014). Our earlier study identified nine PCBs, with PCB118 as the major PCB congener, in the bladders of male pups at postnatal day (P)28–31 following the developmental exposure to the MARBLES mix (0, 0.1, 1, and 6 mg/kg throughout mating, gestation and lactation period), with concentrations 1–2 orders of magnitude lower than the dams in our current study (Keil Stietz et al. 2021). A parallel study that investigated the bladders of pups at P47 ± 4 after developmental exposure to the MARBLES mix identified only 5 PCBs, with an order of PCB153 > PCB180 > PCB118 > PCB138 > PCB28, yet at significantly lower levels (Wang et al. 2026). Comparing the total bladder ƩPCB concentrations across the three studies, for example, in the 6 mg/kg dose group, the ƩPCB were reported as 2650 ng/g in offspring in Keil Stietz et al. (2021), 1800–2200 ng/g in slightly older offspring in Wang et al. (2026), and 30,260 ng/g in the dams in the current study.

**Fig. 2:**
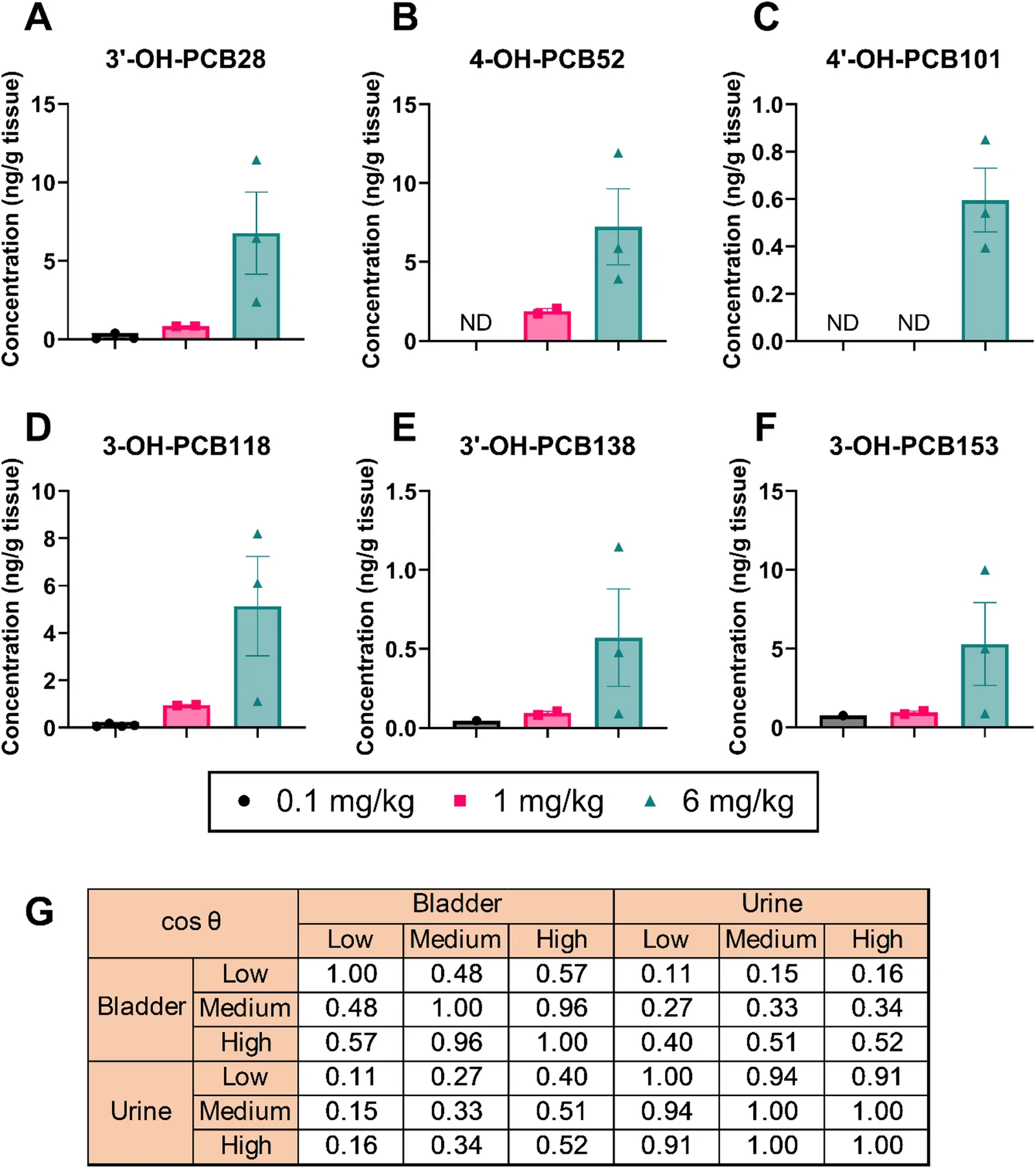
The concentration (ng/g ww) of major OH-PCB congeners, including (**A**) 3’-OH-PCB28, (**B**) 4-OH-PCB52, (**C**) 4’-OH-PCB101, (**D**) 3-OH-PCB118, (**E**) 3’-OH-PCB138, and (**F**) 3-OH-PCB153, in the bladder of post-weaning dams. The profiles of OH-PCB in bladder and urine were compared by the similarity coefficient cos θ (**G**). Due to their small size, bladders were pooled to form pools of approximately 50 mg; *n* = 2–4 pools per exposure group. Each bar was expressed as mean ± SEM, with individual values shown

Twelve OH-PCBs were at detectable levels in at least one of the dosing groups, including 6-OH-PCB11, 5,6-OH-PCB11, 3-OH-PCB28, 3’-OH-PCB28, 5-OH-PCB28, 4-OH-PCB52, 4,5-OH-PCB95, 4’-OH-PCB101, 3-OH-PCB118, 3’-OH-PCB138, 3-OH-PCB153, and 3’-OH-PCB180 (Table S9). The major OH-PCBs included 4-OH-PCB52, 3’-OH-PCB28, 3-OH-PCB118, and 3-OH-PCB153 (Fig. 2, Table S9). Many of them are human relevant metabolites, and are routinely reported in human biospecimens, including 6-OH-PCB11 (Zhu et al. 2013), 3-OH-PCB28, 3’-OH-PCB28 (Quinete et al. 2017), 4-OH-PCB52 (Randerath et al. 2024), 3-OH-PCB118 (Koh et al. 2016), 3’-OH-PCB138 (Marek et al. 2013), 3-OH-PCB153, and 3’-OH-PCB180 (Park et al. 2009). Our earlier study reported fewer, but primarily higher chlorinated metabolites including 3-OH-PCB118, 3’-OH-PCB138, and 3-OH-PCB153 in the bladders of P47 ± 4 pups exposed to the MARBLES mix developmentally through the maternal diet (Wang et al. 2026).

### PCB and OH-PCB levels in the urine

Urine is a route for the excretion of lower-chlorinated PCBs, but not higher-chlorinated PCBs (Birnbaum 1985); however, many PCB disposition studies use metabolic cages. It is unclear whether trace amounts of PCBs in urine reflect urinary excretion or cross-contamination from feces. The present study addresses this uncertainty by collecting free-stream urine. In urine, 8 of the 12 PCB congeners were detected in at least one dose group, in descending order: PCB28 > PCB118 ≈ PCB11 > PCB153 > PCB138 > PCB180 > PCB101 > PCB52 (Table S10).

In contrast to the parent PCBs, urine is a route of elimination of polar PCB metabolites, including PCB sulfates and glucuronides (Dhakal et al. 2013; Grimm et al. 2015). In this study, the total PCB and OH-PCB in urine were 671 ng/g, whereas total levels were 19–81 ng/g in offspring in the high dose group (Wang et al. 2026). After deconjugation, eleven OH-PCBs were at detectable levels in at least one of the dose groups, including 6-OH-PCB11, 2’-OH-PCB28, 5-OH-PCB11, 4-OH-PCB11, 3-OH-PCB28, 3’-OH-PCB28, 5-OH-PCB28, 4-OH-PCB52, 4,5-OH-PCB95, 3-OH-PCB118, and 5-OH-PCB138 (Table S10). The major OH-PCBs included 3’-OH-PCB28, 5-OH-PCB28, and 4-OH-PCB11 (Table S10). According to our recent study, the profiles of PCBs and OH-PCBs in the dams differed from the offspring following developmental exposure to the MARBLES mix. For example, PCB11, PCB52, and their OH-metabolites were not detected in offspring urine (Wang et al. 2026).

### Comparison of PCB and OH-PCB in bladder vs. urine

**Fig. 3:**
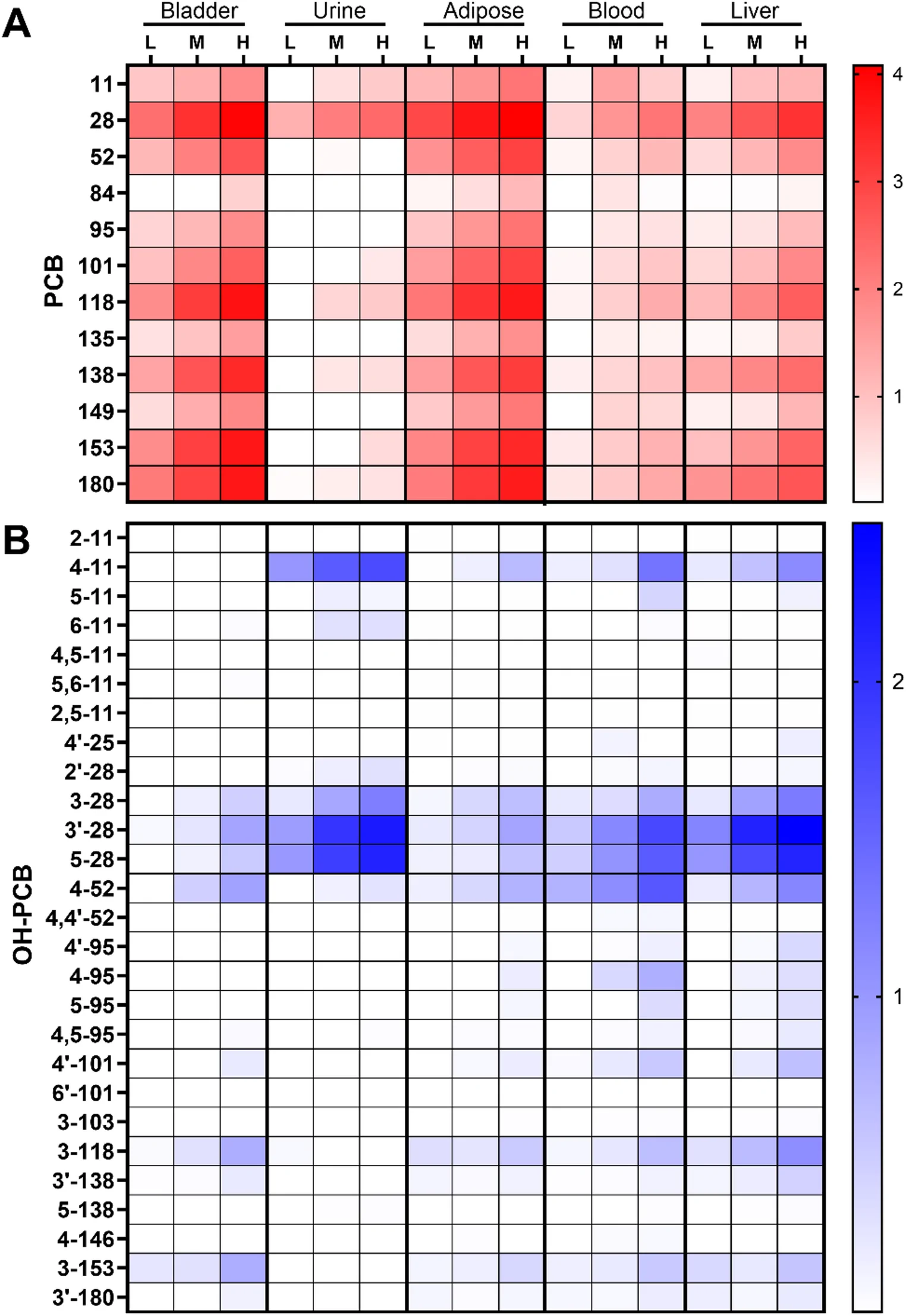
The heatmap compares the levels (ng/g ww) of (**A**) PCBs and (**B**) OH-PCBs in different tissues and urine from post-weaning dams. Values are the log-transformed average. Boxes in white color are below limit of detection (LOD). The abbreviations for OH-PCBs are a short version where the first number indicates the position of the hydroxy group on the biphenyl moiety, and the second number reflects the corresponding PCB congener (e.g., 2–11 indicates 2-OH-PCB11)

When comparing PCB and OH-PCB levels in the bladder and urine, parent PCBs predominate in the bladder, whereas PCB metabolites, measured as OH-PCBs after deconjugation, predominate in urine (Fig. 3). Specifically, OH-PCBs comprise less than 1% of total PCB and OH-PCB in the bladder (Table S9) but account for 60–64% in urine (Table S10). This observation is consistent with urine as a route of excretion for polar, water-soluble PCB metabolites. Importantly, we also observed that bladder tissue and urine have distinct PCB profiles, with a similarity coefficient cos θ of 0.11–0.52 (Fig. 2G), suggesting that the bladder PCB burden is not simply a reflection of urine PCB profiles. The profiles of OH-PCB in bladder and urine were also compared using the similarity coefficient cos θ (Fig. 2G). The cos θ between bladder and urine ranged from 0.11 to 0.52. In contrast, within-tissue comparisons showed higher similarity, ranging from 0.48 to 0.96 in bladder samples and from 0.91 to 1.00 in urine samples.

One possible explanation for the distinct OH-PCB profiles in the bladder versus urine is the presence of a protective urothelial barrier composed of uroplakin proteins, which shield the underlying tissue from urinary compounds (Georgas et al. 2015). This finding also highlights how disease states could impact bladder PCB burden. For example, in states of inflammation or infection, the protective barrier function of the bladder can be compromised (Park et al. 2016; Zwaans et al. 2021; Wu et al. 2022); if this is the case, then it is possible that PCBs or OH-PCBs in the urine could come into contact with the underlying urothelium, stroma, and smooth muscle of the bladder, which could in turn influence bladder function.

One source of the OH-PCB in the bladder could be from the systemic circulation. Although the bladder is not designed for absorption, the urothelium is not completely impermeable. Experimental studies demonstrate that small solutes such as urea and creatinine can be reabsorbed across the bladder epithelium during urine storage (Khandelwal et al. 2009). Accordingly, limited passive diffusion of hydroxylated compounds from urine into urothelial cells may be another source of OH-PCB in bladder tissue. Additionally, the OH-metabolites could be formed locally in the bladder because bladder epithelium expresses CYPs (Wang et al. 2026), a possibility that warrants further investigation.

### Comparison of PCBs and OH-PCBs across tissues

To assess the human relevance of PCB exposures in our current mouse model, we calculated total PCB concentrations in the post-weaning dam blood, which ranged from 10 to 260 ng/g. Using a plasma-to-whole blood distribution coefficient of 1.69 (Batterman et al. 2016) and blood density of 1.057 g/mL (Riches et al. 1973), we estimated that the corresponding serum PCB concentrations range from approximately 18 to 460 ng/g. Although these levels exceed those reported in pregnant women from the MARBLES cohort (0.7 to 12.6 ng/mL) (Sethi et al. 2019), the lowest serum concentration observed in our model is comparable to the upper end of the human exposure range. PCBs and OH-PCBs were also measured in the adipose tissue and liver, compartments for which PCB and metabolite residue data are available. The detection frequencies, profiles, and levels were compared to those observed in the bladder and urine (Fig. 3). All 12 parent PCBs were detected in adipose, blood, and liver (Tables S11-S13). As expected, the liver, as the major site of PCB metabolism (Wahlang et al. 2017), contained the largest number of detectable OH-PCB metabolites, followed by adipose and blood, then bladder and urine. Total PCB concentrations generally followed the order adipose > bladder > liver > urine ≈ blood across most dosing groups, whereas total OH-PCB concentrations were highest in the liver, followed by urine > blood > adipose ≈ bladder (Tables S9-–S13). The bioaccumulation patterns observed for PCBs and OH-PCBs align with their physicochemical characteristics: the greater lipophilicity of PCBs promotes accumulation in fatty tissue, whereas the increased polarity of OH-PCBs facilitates their excretion (Matthews and Dedrick 1984). Both PCBs (Figs. 1 and 3) and OH-PCBs (Figs. 2 and 3) increased with dose in all tissues, although some OH-PCBs deviated from this pattern (e.g., 3′-OH-PCB180 in adipose, blood, and liver). Although the bladder is rarely analyzed in PCB disposition studies, its relatively low PCB burden aligns with previous findings in skeletal muscle (Zhang et al. 2021).

Analysis of individual PCB and OH-PCB metabolites revealed that in adipose, all 12 PCB congeners were detected in a dose-dependent manner, in descending order: PCB28 > PCB118 > PCB180 > PCB153 > PCB138 > PCB52 > PCB101 > PCB95 > PCB11 > PCB149 > PCB135 > PCB84 (Table S11). Twenty-three OH-PCBs were at detectable levels in adipose tissue in at least one dose group, with 3’-OH-PCB28, 3-OH-PCB28, 5-OH-PCB28, 4-OH-PCB52, 3-OH-PCB118, and 4-OH-PCB11 being the major metabolites (Table S11). In blood, all 12 PCB congeners were detected in at least two dose groups, in descending order: PCB28 > PCB180 > PCB118 > PCB153 > PCB52 > PCB138 > PCB101 > PCB11 > PCB149 > PCB95 > PCB135 > PCB84 (Table S12). Twenty-four OH-PCBs were at detectable levels in at least one dose group in blood, with 3’-OH-PCB28, 4-OH-PCB52, 5-OH-PCB28, and 4-OH-PCB11 being major OH-PCBs (Table S12). Finally, in the liver, all 12 PCB congeners were detected in all three dose groups in a dose-dependent manner, in descending order: PCB28 > PCB180 > PCB118 > PCB153 > PCB138 > PCB101 > PCB52 > PCB11 > PCB149 > PCB95 > PCB135 > PCB84 (Table S13). Twenty-five OH-PCBs were at detectable levels in at least one dose group in the liver, with 3’-OH-PCB28 and 5-OH-PCB28 as major OH-PCB congeners (Table S13). The profiles of PCB and OH-PCB are tissue-specific due to the potential differences in the tissue lipophilicity (Li et al. 2024b) and the distribution of cytochrome P450 (CYP) enzymes (Renaud et al. 2011).

### Comparison of individual PCB and OH-PCB congeners across tissues

#### PCB28 and its metabolites

PCB28 was the most abundant congener detected across all tissues. In the high-dose group, concentrations were highest in adipose tissue (12.0 µg/g), followed by bladder (10.2 µg/g), liver (1.8 µg/g), urine (0.25 µg/g), and blood (0.16 µg/g) (Tables S9–S13). This pattern is expected, given that PCB 28 comprises 48.2% of the MARBLES mix (Sethi et al. 2019). Consistent with our findings, previous studies evaluating exposure to the MARBLES mix (6 mg/kg/d) also reported PCB 28 as the predominant congener across multiple tissues, including brain (1.8 µg/g), feces (2.9 µg/g), liver (11.9 µg/g), and serum (1.0 µg/g) of female mice exposed orally to the MARBLES mix for 7 weeks (Dean et al. 2025). However, PCB28 levels in our study were lower than those reported in other studies that used non-pregnant female mice with a similar exposure paradigm. The reduced concentrations observed here likely reflect physiological changes associated with pregnancy and lactation, which are known to alter PCB distribution and burdens in tissues of female mice (Lee et al. 2007; Li et al. 2024a).

PCB28 is metabolized by hepatic CYP enzymes to complex mixtures of OH-PCB metabolites. In this study, 3’-OH-PCB28 was identified as a major metabolite in all tested tissues and matrices, indicating it may be a major metabolite in mice. 5-OH-PCB28 is a major secondary OH-PCB in adipose, blood, liver, and urine. However, a study from an occupationally exposed German cohort has identified 3-OH-PCB28 as the predominant congener in human urine (Quinete et al. 2016), and 3-OH-PCB28 as the most frequently detected metabolite in plasma, while 3’-OH-PCB28 showed the highest concentrations (up to 16.4 ng/mL) in plasma (Quinete et al. 2017). The observed cross-species difference is likely driven by variations in CYP enzyme expression and activity. All the OH-metabolites of PCB28 detected in this study are consistent with the prediction in a toxicokinetic study of PCB28 in rats (Adamu et al. 2025).

#### PCB118 and its metabolites

PCB118 was the second most abundant congener detected in bladder, urine, and adipose, while it was the third most abundant in blood and liver (Table S9-S13). This is in line with previous reports using MARBLES mix exposure in adult mice (Bullert et al. 2024). PCB118 is unique in this mixture is that it is the only dioxin-like PCB present, capable of activating the aryl hydrocarbon receptor (AHR) (Sorg 2014). AHR is present in the bladder, and this pathway has been implicated in bladder carcinogenesis; however, pathway activation can enhance or inhibit tumorigenesis, depending on the tissue type and/or tumor type and stage examined (Safe et al. 2013; Yu et al. 2020). Whether the PCB118 present in the bladder can modulate this pathway alone or influence drug treatments is unknown, but it is of potential clinical significance.

#### PCB11 and its metabolites

PCB11 is an emerging environmental contaminant that differs from legacy PCBs in both its sources and toxicological profile. Unlike historically produced Aroclor mixtures, PCB11 is generated unintentionally during paint pigment manufacturing (Hu and Hornbuckle 2010) and remains widely present in urban air (Vorkamp 2016), leading to continuous human exposure primarily via inhalation. In our study, PCB11 levels were very low, despite PCB11 being the second most abundant congener in the MARBLES mix at 24.3%. This is also consistent with other studies examining PCB11 levels in MARBLES-exposed non-pregnant mice (Bullert et al. 2024; Dean et al. 2025) and is supported by reports of short half-life and rapid biotransformation of PCB11 in rodent exposure models (Hu et al. 2013; Zhang et al. 2021). 4-OH-PCB11 is a major OH-PCB in adipose, blood, liver, and urine, but is not detected in the bladder. This is also confirmed in an inhalation study of PCB11 on rats, which identified 4-OH-PCB11 as the major hydroxylated metabolite in the liver, but not detectable in the lung or serum, due to a fast elimination by further biotransformation (Hu et al. 2013).

### Cyp expression in the bladder

**Fig. 4:**
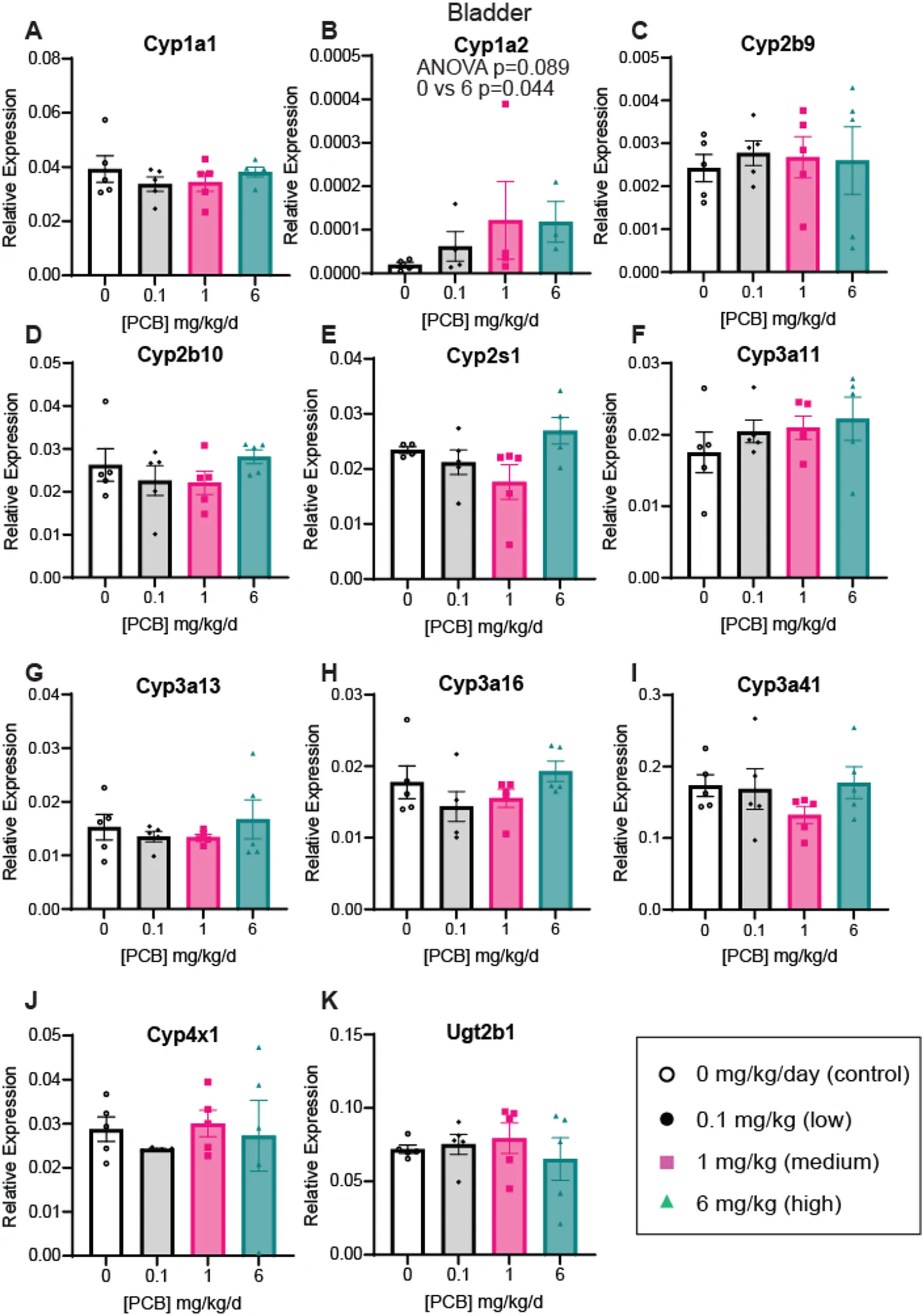
Relative expression of *Cyp* and *Ugt* genes in bladder using RT-qPCR. Following adult exposure to the MARBLES mix, bladders were collected to assess mRNA abundance of *Cyp* and *Ugt* genes. Data are presented as mean ± SEM, *n* = 3–5 mice per group. Data were assessed using one way ANOVA, Welch’s ANOVA or Kruskal Wallis test followed by Dunn’s multiple comparisons test. Prism’s ROUT method was used to identify and remove outliers. p-values < 0.05 were considered significant

The liver is the primary site of PCB metabolism, and PCBs can modify their own metabolism by inducing drug-metabolizing enzymes in the liver (Wahlang et al. 2017). However, the role of extrahepatic metabolism in the disposition of PCBs and their metabolites, especially in pregnant or lactating mice, remains poorly studied. Therefore, we examined whether PCB exposure affected the expression of ten CYP genes and one UDP-glucuronosyltransferase gene in the bladders of exposed dams. Of these genes, *Cyp1a1*, *Cyp2b9*, *Cyp2b10*, *Cyp2s1*, *Cyp3a11*, *Cyp3a13*, *Cyp3a16*, *Cyp3a41*, *Cyp4 × 1*, and *Ugt2b1* showed no change after PCB exposure (Fig. 4). However, for *Cyp1a2*, there was a nearly significant p-value (*p* = 0.089) in the overall ANOVA, and post hoc analysis revealed significance between the 6 mg/kg/d PCB group and the control (*p* = 0.044) (Fig. 4B). In our earlier study, *Cyp* expression showed a significant main effect of sex on the expression of *Cyp1a1*, *Cyp2b9*, *Cyp2b10*, *Cyp2s1*,* Cyp3a11*, *Cyp3a13*, *Cyp3a16*, *Cyp3a41*, *Cyp4 × 1*, and *Ugt2b1* in offspring mice after developmental exposure to MARBLES mix, yet none of the *Cyp* expression was altered by PCBs alone (Wang et al. 2026).

Although we did not investigate *Cyp* activity in this study, our data suggest that PCBs might be locally metabolized in the bladder. Additionally, exposure to PCBs could alter *Cyp1a2* metabolism in the bladder or affect the metabolism of other environmental toxicants or drugs, thereby further influencing disease risk. *Cyp1a2* plays a role in producing reactive metabolites, which are thought to be bladder carcinogens (Bonassi et al. 1989; Landi et al. 1999; Kasperczyk et al. 2025). Moreover, *Cyp1a2* promoter polymorphisms in humans were found to interact with heavy smoking to increase the risk of bladder cancer (Pavanello et al. 2010). *Cyp1a2* is also a downstream target of AHR activation. Given that PCB118, an AHR agonist, was among the most prevalent parent PCBs detected in tissues, it is possible that this PCB congener is responsible for the increase in *Cyp1a2*. Future research should evaluate *Cyp* activity and determine whether increased production of *Cyp1a2*-dependent active metabolites occurs with high PCB exposures and impacts voiding function.

### Cyp expression in the liver

**Fig. 5:**
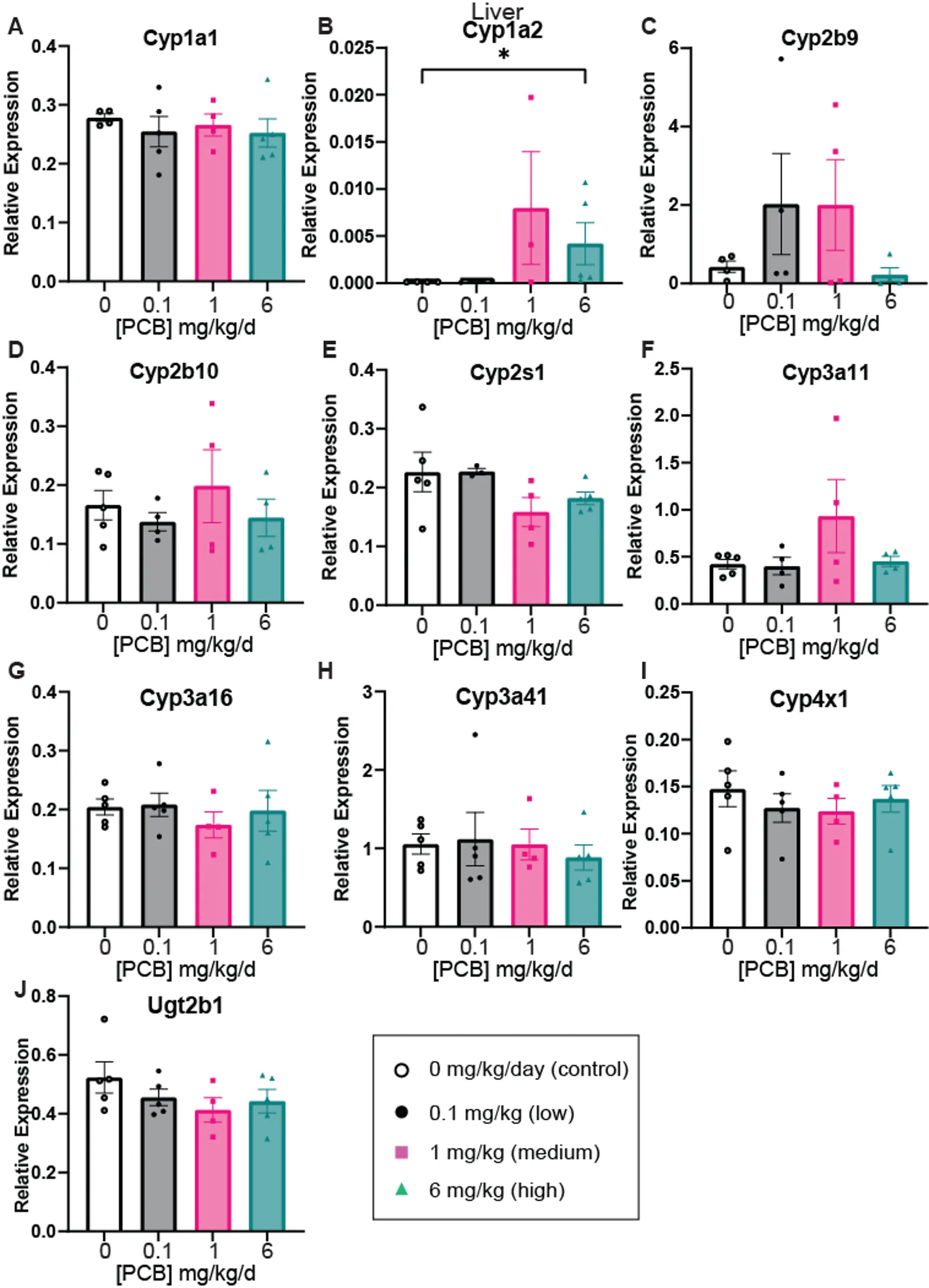
Relative expression of *Cyp* and *Ugt* genes in liver using RT-qPCR. Following adult exposure to the MARBLES mix, livers were collected to assess mRNA abundance of *Cyp* and *Ugt* genes. Data are presented as mean ± SEM, *n* = 3–5 mice per group. Data were assessed using one way ANOVA, Welch’s ANOVA, or Kruskal Wallis test followed by Dunn’s multiple comparisons test. Prism’s ROUT method was used to identify and remove outliers. p-values < 0.05 were considered significant. Bracket and asterisk indicate significant differences between groups

In the liver, similar to the bladder, PCB exposure did not alter expression of *Cyp1a1*, *Cyp2b9*, *Cyp2b10*, *Cyp2s1*, *Cyp3a11*, *Cyp3a16*, *Cyp3a41*, *Cyp4 × 1*, or *Ugt2b1* (Fig. 5). In contrast, *Cyp1a2* expression was significantly increased in the 6 mg/kg/d PCB group relative to vehicle controls (Fig. 5B), indicating that, among the genes examined, *Cyp1a2* is the most responsive to high-dose PCB exposure in the liver of female mice exposed during gestation and lactation. PCBs and other halogenated biphenyls are well-known CYP inducers that act in a structure- and species-dependent manner (Robertson et al. 1984). Dioxin-like congeners activate the aryl hydrocarbon receptor (AHR) and induce Cyp1 family enzymes, whereas non-dioxin-like PCBs preferentially activate the constitutive androstane receptor (CAR) and pregnane X receptor (PXR), leading to induction of Cyp2b and Cyp3a isoforms (Safe 1994). The lack of induction of *Cyp2b* and *Cyp3a* genes in the present study likely reflects the combined influence of PCB mixture composition and a 67-day exposure paradigm, as hepatic CYP responses differ markedly between acute high-dose and chronic low-dose exposures. Acute high-dose exposure typically produces a rapid and robust activation of nuclear receptors, resulting in transient but pronounced induction of CYP isoforms (Konat and Clausen 1973; Robertson et al. 1984). In contrast, chronic low-dose exposure leads to more moderate but sustained CYP induction (Aqil et al. 2014). For example, the commercial PCB mixture Aroclor 1254 has been shown to elevate CYP levels by 360% in mice after 7 days of high-dose dietary exposure (Konat and Clausen 1973). More recent studies using environmentally relevant mixtures support these early studies; oral exposure to the MARBLES mix for 7 weeks produced dose-dependent PCB accumulation in the mouse liver and altered expression of xenobiotic metabolism genes, including *Cyp1a2*, *Cyp2b10*, and *Cyp2c50* (Bullert et al. 2024). Together, these findings underscore the importance of PCB mixture composition, dose, and exposure duration in determining hepatic CYP expression, PCB metabolism, and downstream toxicological outcomes.

## Supplementary Information

Below is the link to the electronic supplementary material.

Supplementary Material 1

## Data Availability

Data availabilityThe data are available from the corresponding author upon reasonable request and at DOI: 10.5061/dryad.gxd25482c.
