# Supplementary material for: Developmental exposure to a human-relevant PCB mixture: impacts on PCB congeners, metabolites, and drug-metabolizing enzymes in the bladder of post-weaning mice
Source: Arch Toxicol. 2026 Jul 4;100(11):5135–50. doi: 10.1007/s00204-026-04498-6 (PMC13637031; doi:10.1007/s00204-026-04498-6)
Supplement: Supplementary file 1 [file 204_2026_4498_MOESM1_ESM.docx]

**Developmental Exposure to a Human-Relevant PCB Mixture: Impacts on PCB Congeners, Metabolites, and Drug-Metabolizing Enzymes in the Bladder of Post-Weaning Mice**

*Hui Wang^1^, Alex Goskowicz^2^, Audrey Spiegelhoff^2^, Conner L. Kennedy^2^, Monica M. Ridlon^2^, Rachel F. Marek^3^, Hans-Joachim Lehmler^1^*, Kimberly P. Keil Stietz^2^**

**Author Affiliation:**

^1^Department of Occupational and Environmental Health, The University of Iowa, Iowa City, IA, 52246, United States of America

^2^Department of Comparative Biosciences, University of Wisconsin-Madison, Madison, WI, 53706, United States of America

^3^Department of Civil & Environmental Engineering, The University of Iowa, Iowa City, IA, 52246, United States of America

**ORCID:**

Hui Wang: <https://orcid.org/0000-0002-5132-0077>

Alex Goskowicz: <https://orcid.org/0009-0006-3568-1477>

Audrey Spiegelhoff: <https://orcid.org/0000-0002-8073-4535>

Conner L. Kennedy: <https://orcid.org/0000-0003-3104-6688>

Monica M. Ridlon: <https://orcid.org/0000-0001-8605-4453>

Rachel F. Marek: <https://orcid.org/0000-0002-7898-2900>

Kimberly P. Keil Stietz: <https://orcid.org/0000-0002-7006-9420>

Hans-Joachim Lehmler: <https://orcid.org/0000-0001-9163-927X>

***Co-Corresponding Author:**

Dr. Kimberly Keil Stietz

University of Wisconsin-Madison

Department of Comparative Biosciences

School of Veterinary Medicine

2015 Linden Dr. Madison, WI 53706

Phone: (608)-265-2879

e-mail: [kkeil@wisc.edu](mailto:kkeil@wisc.edu)

Dr. Hans-Joachim Lehmler

The University of Iowa

Department of Occupational and Environmental Health

UI Research Park, #221 IREH, Iowa City, IA 52242-5000

Phone: (319) 335-4310

e-mail: [hans-joachim-lehmler@uiowa.edu](mailto:hans-joachim-lehmler@uiowa.edu)

**Acknowledgments**

The study was supported by grants P30 ES005605 from the Environmental Health Sciences Research Center and P43 ES013661 from the Iowa Superfund Research Program, which funded the authentication of the test compounds and the analytical work. Animal generation and qPCR analysis were supported by the National Institutes of Health, specifically the NIEHS R01 ES035020 and R00 ES029537 to KPKS, F31 ES036876 to MMR, and T32 ES007015 to both MMR and CLK. The content is solely the responsibility of the authors and does not necessarily represent the official views of the National Institute of Environmental Health Sciences or the National Institutes of Health **Table of Contents**

| Chemicals and Analytical Standards | S3 |
| --- | --- |
| PCB and OH-PCB Extraction from Dam Tissue, Blood, and Urine | S4 |
| GC-MS/MS Instrumental Parameters | S5 |
| **Table S1.** The canonical SMILES structure of all 209 PCB congeners. | S7 |
| **Table S2.** The canonical SMILES structure of the 124 available methoxylated PCBs used as calibration standards for corresponding OH-PCBs. | S17 |
| **Table S3.** Body mass (g) of dams used in this study is unaltered by PCBs. | S23 |
| **Table S4.** Precursor ions, product ions, and collision energies for each analyte in the GC-MS/MS analysis. | S24 |
| **Table S5.** Ongoing Precision Recovery (OPR) for selected PCBs and OH-PCBs in method blanks and tissue matrices. | S26 |
| **Table S6.** Recoveries (%) of surrogate standards for PCB and OH-PCB in different biological matrices. | S27 |
| **Table S7.** Method Detection Limits (MDLs) and Limits of Detection (LODs) for PCBs from MARBLES mixture and their possible OH-PCB metabolites. | S28 |
| **Table S8.** Table of primers used for qPCR. | S30 |
| **Table S9**. The levels of PCB and OH-PCB (ng/g ww tissue) in bladder from female dam exposed via diet to 0.1, 1, or 6 mg/kg body weight of the MARBLES PCB mixture. | S31 |
| **Table S10**. The levels of PCB and OH-PCB (ng/g ww) in urine from female dam exposed via diet to 0.1, 1, or 6 mg/kg body weight of the MARBLES PCB mixture. | S33 |
| **Table S11**. The levels of PCB and OH-PCB (ng/g ww tissue) in adipose tissue from female dam exposed via diet to 0.1, 1, or 6 mg/kg body weight of the MARBLES PCB mixture | S35 |
| **Table S12**. The levels of PCB and OH-PCB (ng/g ww) in blood from female dam exposed via diet to 0.1, 1, or 6 mg/kg body weight of the MARBLES PCB mixture. | S37 |
| **Table S13**. The levels of PCB and OH-PCB (ng/g ww tissue) in the liver from female dam exposed via diet to 0.1, 1, or 6 mg/kg body weight of the MARBLES PCB mixture. | S39 |
|  |  |

**Chemicals and Analytical Standards**

All solvents (pesticide grade), sulfuric acid (concentrated), hydrochloric acid (concentrated), potassium chloride, sodium chloride, silica gel, and sodium sulfite were purchased from Fisher Scientific (Fair Lawn, NJ, USA). Tetrabutylammonium hydrogen sulfate was obtained from J.T. Baker (Phillipsburg, NJ, USA). A solution of diazomethane in diethyl ether was synthesized using an Aldrich Mini Diazald® Apparatus as described.(Black, 1983) Individual PCB and OH-PCB standards were purchased from AccuStandard (New Haven, CT, USA) unless otherwise stated. 2,5-Dichlorobiphenyl-4'-ol (4'-OH-PCB9), 3,3'-dichlorobiphenyl-4-ol (4-OH-PCB11), 2,3',4-trichlorobiphenyl-4'-ol (4'-OH-PCB25), 2,2',5,5'-tetrachlorobiphenyl-4-ol (4-OH-PCB52), 2,2',3,4',6-pentachlorobiphenyl-4-ol (4-OH-PCB91), 2,2',3,5',6-pentachlorobiphenyl-4-ol (4-OH-PCB95), and 2,2',3,5',6-pentachlorobiphenyl-5-ol (5-OH-PCB95) were prepared as described.(Lehmler and Robertson, 2001a; Li et al., 2010; Joshi et al., 2011; Rodriguez et al., 2016; Alam et al., 2018) 4,4'-Dichlorobiphenyl (PCB15) and 2,3,4',5,6-pentachlorobiphenyl (PCB117) (100 ng/mL each in isooctane) were used as surrogate standards for PCBs, and 4'-OH-PCB9, 4-OH-PCB91, and 2,3,3',4,5,5'-hexachlorobiphenyl-4'-ol (4'-OH-PCB159) (100 ng/mL each in methanol) were used as surrogate standards for OH-PCBs. Deuterium-labeled 2,4,6-trichlorobiphenyl-2',3',4',5',6'-d_5_ (d-PCB30; CDN Isotopes, Quebec, Canada) and 2,2',3,4,4',5,6,6'-octachlorobiphenyl (PCB204) were used as internal standards (volume correctors).

Several calibration standards were prepared for peak verification and mass calibration. A PCB calibration standard was prepared from standard mixtures containing all 209 PCB congeners (AccuStandard). Two MeO-PCB calibration standard solutions were used for the analysis of MeO-PCBs. MeO-PCB standard solution 1 contained seventy-two methoxylated PCBs (MeO-PCBs) (70 mono-MeO-PCBs and two di-MeO-PCBs) provided by AccuStandard or Wellington Laboratories (Guelph, ON, Canada). MeO-PCB standard solution 2 consisted of fifty-two MeO-PCBs (28 mono-MeO-PCBs and 24 di-MeO-PCBs as standard Solution 2). These MeO-PCBs were synthesized with Suzuki coupling reaction between a suitable benzene boronic acid and a methoxylated bromochlorobenzene (Lehmler and Robertson, 2001a, b) and authenticated as described.(McLean et al., 1996; Zhai et al., 2011; Rodriguez et al., 2016; Li et al., 2018; Dhakal et al., 2020; Saktrakulkla et al., 2020). In addition, an OH-PCB standard solution was prepared containing 4-OH-PCB11, 4'-OH-PCB25, 4-OH-PCB52, 4-OH-PCB95, 5-OH-PCB95, 2,2',4,4',5,5'-hexachlorobiphenyl-3-ol (3-OH-PCB153), and 2,2',3,4',5,5'-hexachlorobiphenyl-4-ol (4-OH-PCB146) in methanol (100 ng/mL each).

**PCB and OH-PCB Extraction from Dam Tissue, Blood, and Urine**

A liquid-liquid extraction method was used to simultaneously extract and analyze PCBs and OH-PCBs. Briefly, liver (28±4 mg, n=5-6 mice/exposure group), adipose tissue (143±18 mg, n=5-6 mice/exposure group), or pooled bladders (45±8 mg, n=2-4 pools/exposure group) were homogenized with 3 mL 2-propanol using a TissueRuptor (QIAGEN, Hilden, Germany). After spiking 10 ng PCB15 and PCB117 and 10 ng OH-PCB (4'-OH-PCB9, 4-OH-PCB91, and 4'-OH-PCB159) as surrogate standards to all samples, PCBs and OH-PCBs were extracted with diethyl ether and hexane (1:9, v/v), followed by washing the organic extracts with 5 mL of 0.1 M phosphoric acid in 0.9% sodium chloride solution. The extracts were concentrated under a gentle stream of nitrogen and derivatized with diazomethane at 4°C overnight.(Kania-Korwel et al., 2008) The extracts were passed through a sulfuric acid and silica gel (1:2, w/w) cartridge for further cleanup. Finally, the extracts were concentrated under gentle nitrogen flow and spiked with the internal standard (d-PCB30 and PCB204) for gas chromatography with tandem mass spectrometry (GC-MS/MS) analysis.

Blood samples (415±15 mg, n=5-6 mice/exposure group) were extracted similarly, but with slight modifications. Briefly, 1 mL of 6 M HCl was added to the serum. After spiking the surrogate standard, PCB and OH-PCB were extracted using a 2-propanol and hexane: methyl tert-butyl ether (MTBE) (1:1, v/v) mixture. After washing with 3 mL 1% KCl, the extracts were concentrated and derivatized as described above. After further cleanup with 2-propanol and tetrabutylammonium hydrogen sulfate (TBA), the extracts were subjected to the same cleanup steps as described for the extraction of bladder tissue.

Twenty µL of sulfatase (type H-2 from *Helix pomatia*, Sigma-Aldrich, St. Louis, MO, USA) was added to pooled urine samples (282±146 mg, n=5-6 mice/exposure group) to convert PCB conjugates to OH-PCBs in a shaking water bath for 16 h at 37 °C.(Dhakal et al., 2012) The extraction of PCBs and OH-PCBs in urine samples after deconjugation followed the same procedure as described above for blood extraction.

**GC-MS/MS Instrumental Parameters**

PCBs and OH-PCBs were quantified by a GC-MS/MS system (Agilent 7890B GC system, Agilent 7000D Triple Quad, Agilent 7693 autosampler) equipped with an SPB-Octyl capillary column (50% n-octyl/50% methyl siloxane, 30 m length, 0.25 mm inner diameter, 0.25 µm film thickness; Supelco, Bellefonte, PA).(Wang et al., 2023) Helium was used as the carrier gas (0.8 mL/min), and nitrogen was used as the collision gas. The gas chromatograph was operated in solvent vent injection mode with the following setup: initial temperature, 45 °C; initial time, 0.06 min; ramp, 600 °C/min to inlet temperature of 325 °C at 5 psi. The oven temperature program of the gas chromatograph was 45 °C for 2 min, 45 to 75 °C at 100 °C/min, hold for 5 min, 75 to 150 °C at 15 °C/min, hold for 1 min, 150 to 280 at 2.5 °C/min, and hold 5 min. The triple quadrupole electron ionization source was set to 230 °C. Congener mass was quantified by applying a relative response factor obtained from the calibration standard for each congener (Marek et al., 2013).

**Table S1.** The canonical SMILES structure of all 209 PCB congeners. The molecular structure and IUPAC names are also listed for comparison.

| **EPA Name** | **Canonical SMILES** | **IUPAC Name** |
| --- | --- | --- |
| PCB1 | C1=CC=C(C=C1)C2=CC=CC=C2Cl | 2-Chloro-1,1'-biphenyl |
| PCB2 | C1=CC=C(C=C1)C2=CC(=CC=C2)Cl | 3-Chloro-1,1'-biphenyl |
| PCB3 | C1=CC=C(C=C1)C2=CC=C(C=C2)Cl | 4-Chloro-1,1'-biphenyl |
| PCB4 | C1=CC=C(C(=C1)C2=CC=CC=C2Cl)Cl | 2,2'-Dichloro-1,1'-biphenyl |
| PCB5 | C1=CC=C(C=C1)C2=C(C(=CC=C2)Cl)Cl | 2,3-Dichloro-1,1'-biphenyl |
| PCB6 | C1=CC=C(C(=C1)C2=CC(=CC=C2)Cl)Cl | 2,3'-Dichloro-1,1'-biphenyl |
| PCB7 | C1=CC=C(C=C1)C2=C(C=C(C=C2)Cl)Cl | 2,4-Dichloro-1,1'-biphenyl |
| PCB8 | C1=CC=C(C(=C1)C2=CC=C(C=C2)Cl)Cl | 2,4'-Dichloro-1,1'-biphenyl |
| PCB9 | C1=CC=C(C=C1)C2=C(C=CC(=C2)Cl)Cl | 2,5-Dichloro-1,1'-biphenyl |
| PCB10 | C1=CC=C(C=C1)C2=C(C=CC=C2Cl)Cl | 2,6-Dichloro-1,1'-biphenyl |
| PCB11 | C1=CC(=CC(=C1)Cl)C2=CC(=CC=C2)Cl | 3,3'-Dichloro-1,1'-biphenyl |
| PCB12 | C1=CC=C(C=C1)C2=CC(=C(C=C2)Cl)Cl | 3,4-Dichloro-1,1'-biphenyl |
| PCB13 | C1=CC(=CC(=C1)Cl)C2=CC=C(C=C2)Cl | 3,4'-Dichloro-1,1'-biphenyl |
| PCB14 | C1=CC=C(C=C1)C2=CC(=CC(=C2)Cl)Cl | 3,5-Dichloro-1,1'-biphenyl |
| PCB15 | C1=CC(=CC=C1C2=CC=C(C=C2)Cl)Cl | 4,4'-Dichloro-1,1'-biphenyl |
| PCB16 | C1=CC=C(C(=C1)C2=C(C(=CC=C2)Cl)Cl)Cl | 2,2',3-Trichloro-1,1'-biphenyl |
| PCB17 | C1=CC=C(C(=C1)C2=C(C=C(C=C2)Cl)Cl)Cl | 2,2',4-Trichloro-1,1'-biphenyl |
| PCB18 | C1=CC=C(C(=C1)C2=C(C=CC(=C2)Cl)Cl)Cl | 2,2',5-Trichloro-1,1'-biphenyl |
| PCB19 | C1=CC=C(C(=C1)C2=C(C=CC=C2Cl)Cl)Cl | 2,2',6-Trichloro-1,1'-biphenyl |
| PCB20 | C1=CC(=CC(=C1)Cl)C2=C(C(=CC=C2)Cl)Cl | 2,3,3'-Trichloro-1,1'-biphenyl |
| PCB21 | C1=CC=C(C=C1)C2=C(C(=C(C=C2)Cl)Cl)Cl | 2,3,4-Trichloro-1,1'-biphenyl |
| PCB22 | C1=CC(=C(C(=C1)Cl)Cl)C2=CC=C(C=C2)Cl | 2,3,4'-Trichloro-1,1'-biphenyl |
| PCB23 | C1=CC=C(C=C1)C2=C(C(=CC(=C2)Cl)Cl)Cl | 2,3,5-Trichloro-1,1'-biphenyl |
| PCB24 | C1=CC=C(C=C1)C2=C(C=CC(=C2Cl)Cl)Cl | 2,3,6-Trichloro-1,1'-biphenyl |
| PCB25 | C1=CC(=CC(=C1)Cl)C2=C(C=C(C=C2)Cl)Cl | 2,3',4-Trichloro-1,1'-biphenyl |
| PCB26 | C1=CC(=CC(=C1)Cl)C2=C(C=CC(=C2)Cl)Cl | 2,3',5-Trichloro-1,1'-biphenyl |
| PCB27 | C1=CC(=CC(=C1)Cl)C2=C(C=CC=C2Cl)Cl | 2,3',6-Trichloro-1,1'-biphenyl |
| PCB28 | C1=CC(=CC=C1C2=C(C=C(C=C2)Cl)Cl)Cl | 2,4,4'-Trichloro-1,1'-biphenyl |
| PCB29 | C1=CC=C(C=C1)C2=CC(=C(C=C2Cl)Cl)Cl | 2,4,5-Trichloro-1,1'-biphenyl |
| PCB30 | C1=CC=C(C=C1)C2=C(C=C(C=C2Cl)Cl)Cl | 2,4,6-Trichloro-1,1'-biphenyl |
| PCB31 | C1=CC(=CC=C1C2=C(C=CC(=C2)Cl)Cl)Cl | 2,4',5-Trichloro-1,1'-biphenyl |
| PCB32 | C1=CC(=C(C(=C1)Cl)C2=CC=C(C=C2)Cl)Cl | 2,4',6-Trichloro-1,1'-biphenyl |
| PCB33 | C1=CC=C(C(=C1)C2=CC(=C(C=C2)Cl)Cl)Cl | 2,3',4'-Trichloro-1,1'-biphenyl |
| PCB34 | C1=CC=C(C(=C1)C2=CC(=CC(=C2)Cl)Cl)Cl | 2,3',5'-Trichloro-1,1'-biphenyl |
| PCB35 | C1=CC(=CC(=C1)Cl)C2=CC(=C(C=C2)Cl)Cl | 3,3',4-Trichloro-1,1'-biphenyl |
| PCB36 | C1=CC(=CC(=C1)Cl)C2=CC(=CC(=C2)Cl)Cl | 3,3',5-Trichloro-1,1'-biphenyl |
| PCB37 | C1=CC(=CC=C1C2=CC(=C(C=C2)Cl)Cl)Cl | 3,4,4'-Trichloro-1,1'-biphenyl |
| PCB38 | C1=CC=C(C=C1)C2=CC(=C(C(=C2)Cl)Cl)Cl | 3,4,5-Trichloro-1,1'-biphenyl |
| PCB39 | C1=CC(=CC=C1C2=CC(=CC(=C2)Cl)Cl)Cl | 3,4',5-Trichloro-1,1'-biphenyl |
| PCB40 | C1=CC(=C(C(=C1)Cl)Cl)C2=C(C(=CC=C2)Cl)Cl | 2,2',3,3'-Tetrachloro-1,1'-biphenyl |
| PCB41 | C1=CC=C(C(=C1)C2=C(C(=C(C=C2)Cl)Cl)Cl)Cl | 2,2',3,4-Tetrachloro-1,1'-biphenyl |
| PCB42 | C1=CC(=C(C(=C1)Cl)Cl)C2=C(C=C(C=C2)Cl)Cl | 2,2',3,4'-Tetrachloro-1,1'-biphenyl |
| PCB43 | C1=CC=C(C(=C1)C2=C(C(=CC(=C2)Cl)Cl)Cl)Cl | 2,2',3,5-Tetrachloro-1,1'-biphenyl |
| PCB44 | C1=CC(=C(C(=C1)Cl)Cl)C2=C(C=CC(=C2)Cl)Cl | 2,2',3,5'-Tetrachloro-1,1'-biphenyl |
| PCB45 | C1=CC=C(C(=C1)C2=C(C=CC(=C2Cl)Cl)Cl)Cl | 2,2',3,6-Tetrachloro-1,1'-biphenyl |
| PCB46 | C1=CC(=C(C(=C1)Cl)Cl)C2=C(C=CC=C2Cl)Cl | 2,2',3,6'-Tetrachloro-1,1'-biphenyl |
| PCB47 | C1=CC(=C(C=C1Cl)Cl)C2=C(C=C(C=C2)Cl)Cl | 2,2',4,4'-Tetrachloro-1,1'-biphenyl |
| PCB48 | C1=CC=C(C(=C1)C2=CC(=C(C=C2Cl)Cl)Cl)Cl | 2,2',4,5-Tetrachloro-1,1'-biphenyl |
| PCB49 | C1=CC(=C(C=C1Cl)Cl)C2=C(C=CC(=C2)Cl)Cl | 2,2',4,5'-Tetrachloro-1,1'-biphenyl |
| PCB50 | C1=CC=C(C(=C1)C2=C(C=C(C=C2Cl)Cl)Cl)Cl | 2,2',4,6-Tetrachloro-1,1'-biphenyl |
| PCB51 | C1=CC(=C(C(=C1)Cl)C2=C(C=C(C=C2)Cl)Cl)Cl | 2,2',4,6'-Tetrachloro-1,1'-biphenyl |
| PCB52 | C1=CC(=C(C=C1Cl)C2=C(C=CC(=C2)Cl)Cl)Cl | 2,2',5,5'-Tetrachloro-1,1'-biphenyl |
| PCB53 | C1=CC(=C(C(=C1)Cl)C2=C(C=CC(=C2)Cl)Cl)Cl | 2,2',5,6'-Tetrachloro-1,1'-biphenyl |
| PCB54 | C1=CC(=C(C(=C1)Cl)C2=C(C=CC=C2Cl)Cl)Cl | 2,2',6,6'-Tetrachloro-1,1'-biphenyl |
| PCB55 | C1=CC(=CC(=C1)Cl)C2=C(C(=C(C=C2)Cl)Cl)Cl | 2,3,3',4-Tetrachloro-1,1'-biphenyl |
| PCB56 | C1=CC(=C(C(=C1)Cl)Cl)C2=CC(=C(C=C2)Cl)Cl | 2,3,3',4'-Tetrachloro-1,1'-biphenyl |
| PCB57 | C1=CC(=CC(=C1)Cl)C2=C(C(=CC(=C2)Cl)Cl)Cl | 2,3,3',5-Tetrachloro-1,1'-biphenyl |
| PCB58 | C1=CC(=C(C(=C1)Cl)Cl)C2=CC(=CC(=C2)Cl)Cl | 2,3,3',5'-Tetrachloro-1,1'-biphenyl |
| PCB59 | C1=CC(=CC(=C1)Cl)C2=C(C=CC(=C2Cl)Cl)Cl | 2,3,3',6-Tetrachloro-1,1'-biphenyl |
| PCB60 | C1=CC(=CC=C1C2=C(C(=C(C=C2)Cl)Cl)Cl)Cl | 2,3,4,4'-Tetrachloro-1,1'-biphenyl |
| PCB61 | C1=CC=C(C=C1)C2=CC(=C(C(=C2Cl)Cl)Cl)Cl | 2,3,4,5-Tetrachloro-1,1'-biphenyl |
| PCB62 | C1=CC=C(C=C1)C2=C(C(=C(C=C2Cl)Cl)Cl)Cl | 2,3,4,6-Tetrachloro-1,1'-biphenyl |
| PCB63 | C1=CC(=CC=C1C2=C(C(=CC(=C2)Cl)Cl)Cl)Cl | 2,3,4',5-Tetrachloro-1,1'-biphenyl |
| PCB64 | C1=CC(=CC=C1C2=C(C=CC(=C2Cl)Cl)Cl)Cl | 2,3,4',6-Tetrachloro-1,1'-biphenyl |
| PCB65 | C1=CC=C(C=C1)C2=C(C(=CC(=C2Cl)Cl)Cl)Cl | 2,3,5,6-Tetrachloro-1,1'-biphenyl |
| PCB66 | C1=CC(=C(C=C1C2=C(C=C(C=C2)Cl)Cl)Cl)Cl | 2,3',4,4'-Tetrachloro-1,1'-biphenyl |
| PCB67 | C1=CC(=CC(=C1)Cl)C2=CC(=C(C=C2Cl)Cl)Cl | 2,3',4,5-Tetrachloro-1,1'-biphenyl |
| PCB68 | C1=CC(=C(C=C1Cl)Cl)C2=CC(=CC(=C2)Cl)Cl | 2,3',4,5'-Tetrachloro-1,1'-biphenyl |
| PCB69 | C1=CC(=CC(=C1)Cl)C2=C(C=C(C=C2Cl)Cl)Cl | 2,3',4,6-Tetrachloro-1,1'-biphenyl |
| PCB70 | C1=CC(=C(C=C1C2=C(C=CC(=C2)Cl)Cl)Cl)Cl | 2,3',4',5-Tetrachloro-1,1'-biphenyl |
| PCB71 | C1=CC(=C(C(=C1)Cl)C2=CC(=C(C=C2)Cl)Cl)Cl | 2,3',4',6-Tetrachloro-1,1'-biphenyl |
| PCB72 | C1=CC(=C(C=C1Cl)C2=CC(=CC(=C2)Cl)Cl)Cl | 2,3',5,5'-Tetrachloro-1,1'-biphenyl |
| PCB73 | C1=CC(=C(C(=C1)Cl)C2=CC(=CC(=C2)Cl)Cl)Cl | 2,3',5',6-Tetrachloro-1,1'-biphenyl |
| PCB74 | C1=CC(=CC=C1C2=CC(=C(C=C2Cl)Cl)Cl)Cl | 2,4,4',5-Tetrachloro-1,1'-biphenyl |
| PCB75 | C1=CC(=CC=C1C2=C(C=C(C=C2Cl)Cl)Cl)Cl | 2,4,4',6-Tetrachloro-1,1'-biphenyl |
| PCB76 | C1=CC=C(C(=C1)C2=CC(=C(C(=C2)Cl)Cl)Cl)Cl | 2,3',4',5'-Tetrachloro-1,1'-biphenyl |
| PCB77 | C1=CC(=C(C=C1C2=CC(=C(C=C2)Cl)Cl)Cl)Cl | 3,3',4,4'-Tetrachloro-1,1'-biphenyl |
| PCB78 | C1=CC(=CC(=C1)Cl)C2=CC(=C(C(=C2)Cl)Cl)Cl | 3,3',4,5-Tetrachloro-1,1'-biphenyl |
| PCB79 | C1=CC(=C(C=C1C2=CC(=CC(=C2)Cl)Cl)Cl)Cl | 3,3',4,5'-Tetrachloro-1,1'-biphenyl |
| PCB80 | C1=C(C=C(C=C1Cl)Cl)C2=CC(=CC(=C2)Cl)Cl | 3,3',5,5'-Tetrachloro-1,1'-biphenyl |
| PCB81 | C1=CC(=CC=C1C2=CC(=C(C(=C2)Cl)Cl)Cl)Cl | 3,4,4',5-Tetrachloro-1,1'-biphenyl |
| PCB82 | C1=CC(=C(C(=C1)Cl)Cl)C2=C(C(=C(C=C2)Cl)Cl)Cl | 2,2',3,3',4-Pentachloro-1,1'-biphenyl |
| PCB83 | C1=CC(=C(C(=C1)Cl)Cl)C2=C(C(=CC(=C2)Cl)Cl)Cl | 2,2',3,3',5-Pentachloro-1,1'-biphenyl |
| PCB84 | C1=CC(=C(C(=C1)Cl)Cl)C2=C(C=CC(=C2Cl)Cl)Cl | 2,2',3,3',6-Pentachloro-1,1'-biphenyl |
| PCB85 | C1=CC(=C(C=C1Cl)Cl)C2=C(C(=C(C=C2)Cl)Cl)Cl | 2,2',3,4,4'-Pentachloro-1,1'-biphenyl |
| PCB86 | C1=CC=C(C(=C1)C2=CC(=C(C(=C2Cl)Cl)Cl)Cl)Cl | 2,2',3,4,5-Pentachloro-1,1'-biphenyl |
| PCB87 | C1=CC(=C(C=C1Cl)C2=C(C(=C(C=C2)Cl)Cl)Cl)Cl | 2,2',3,4,5'-Pentachloro-1,1'-biphenyl |
| PCB88 | C1=CC=C(C(=C1)C2=C(C(=C(C=C2Cl)Cl)Cl)Cl)Cl | 2,2',3,4,6-Pentachloro-1,1'-biphenyl |
| PCB89 | C1=CC(=C(C(=C1)Cl)C2=C(C(=C(C=C2)Cl)Cl)Cl)Cl | 2,2',3,4,6'-Pentachloro-1,1'-biphenyl |
| PCB90 | C1=CC(=C(C=C1Cl)Cl)C2=C(C(=CC(=C2)Cl)Cl)Cl | 2,2',3,4',5-Pentachloro-1,1'-biphenyl |
| PCB91 | C1=CC(=C(C=C1Cl)Cl)C2=C(C=CC(=C2Cl)Cl)Cl | 2,2',3,4',6-Pentachloro-1,1'-biphenyl |
| PCB92 | C1=CC(=C(C=C1Cl)C2=C(C(=CC(=C2)Cl)Cl)Cl)Cl | 2,2',3,5,5'-Pentachloro-1,1'-biphenyl |
| PCB93 | C1=CC=C(C(=C1)C2=C(C(=CC(=C2Cl)Cl)Cl)Cl)Cl | 2,2',3,5,6-Pentachloro-1,1'-biphenyl |
| PCB94 | C1=CC(=C(C(=C1)Cl)C2=C(C(=CC(=C2)Cl)Cl)Cl)Cl | 2,2',3,5,6'-Pentachloro-1,1'-biphenyl |
| PCB95 | C1=CC(=C(C=C1Cl)C2=C(C=CC(=C2Cl)Cl)Cl)Cl | 2,2',3,5',6-Pentachloro-1,1'-biphenyl |
| PCB96 | C1=CC(=C(C(=C1)Cl)C2=C(C=CC(=C2Cl)Cl)Cl)Cl | 2,2',3,6,6'-Pentachloro-1,1'-biphenyl |
| PCB97 | C1=CC(=C(C(=C1)Cl)Cl)C2=CC(=C(C=C2Cl)Cl)Cl | 2,2',3,4',5'-Pentachloro-1,1'-biphenyl |
| PCB98 | C1=CC(=C(C(=C1)Cl)Cl)C2=C(C=C(C=C2Cl)Cl)Cl | 2,2',3,4',6'-Pentachloro-1,1'-biphenyl |
| PCB99 | C1=CC(=C(C=C1Cl)Cl)C2=CC(=C(C=C2Cl)Cl)Cl | 2,2',4,4',5-Pentachloro-1,1'-biphenyl |
| PCB100 | C1=CC(=C(C=C1Cl)Cl)C2=C(C=C(C=C2Cl)Cl)Cl | 2,2',4,4',6-Pentachloro-1,1'-biphenyl |
| PCB101 | C1=CC(=C(C=C1Cl)C2=CC(=C(C=C2Cl)Cl)Cl)Cl | 2,2',4,5,5'-Pentachloro-1,1'-biphenyl |
| PCB102 | C1=CC(=C(C(=C1)Cl)C2=CC(=C(C=C2Cl)Cl)Cl)Cl | 2,2',4,5,6'-Pentachloro-1,1'-biphenyl |
| PCB103 | C1=CC(=C(C=C1Cl)C2=C(C=C(C=C2Cl)Cl)Cl)Cl | 2,2',4,5',6-Pentachloro-1,1'-biphenyl |
| PCB104 | C1=CC(=C(C(=C1)Cl)C2=C(C=C(C=C2Cl)Cl)Cl)Cl | 2,2',4,6,6'-Pentachloro-1,1'-biphenyl |
| PCB105 | C1=CC(=C(C=C1C2=C(C(=C(C=C2)Cl)Cl)Cl)Cl)Cl | 2,3,3',4,4'-Pentachloro-1,1'-biphenyl |
| PCB106 | C1=CC(=CC(=C1)Cl)C2=CC(=C(C(=C2Cl)Cl)Cl)Cl | 2,3,3',4,5-Pentachloro-1,1'-biphenyl |
| PCB107 | C1=CC(=C(C=C1C2=C(C(=CC(=C2)Cl)Cl)Cl)Cl)Cl | 2,3,3',4',5-Pentachloro-1,1'-biphenyl |
| PCB108 | C1=CC(=C(C(=C1C2=CC(=CC(=C2)Cl)Cl)Cl)Cl)Cl | 2,3,3',4,5'-Pentachloro-1,1'-biphenyl |
| PCB109 | C1=CC(=CC(=C1)Cl)C2=C(C(=C(C=C2Cl)Cl)Cl)Cl | 2,3,3',4,6-Pentachloro-1,1'-biphenyl |
| PCB110 | C1=CC(=C(C=C1C2=C(C=CC(=C2Cl)Cl)Cl)Cl)Cl | 2,3,3',4',6-Pentachloro-1,1'-biphenyl |
| PCB111 | C1=C(C=C(C=C1Cl)Cl)C2=C(C(=CC(=C2)Cl)Cl)Cl | 2,3,3',5,5'-Pentachloro-1,1'-biphenyl |
| PCB112 | C1=CC(=CC(=C1)Cl)C2=C(C(=CC(=C2Cl)Cl)Cl)Cl | 2,3,3',5,6-Pentachloro-1,1'-biphenyl |
| PCB113 | C1=CC(=C(C(=C1Cl)C2=CC(=CC(=C2)Cl)Cl)Cl)Cl | 2,3,3',5',6-Pentachloro-1,1'-biphenyl |
| PCB114 | C1=CC(=CC=C1C2=CC(=C(C(=C2Cl)Cl)Cl)Cl)Cl | 2,3,4,4',5-Pentachloro-1,1'-biphenyl |
| PCB115 | C1=CC(=CC=C1C2=C(C(=C(C=C2Cl)Cl)Cl)Cl)Cl | 2,3,4,4',6-Pentachloro-1,1'-biphenyl |
| PCB116 | C1=CC=C(C=C1)C2=C(C(=C(C(=C2Cl)Cl)Cl)Cl)Cl | 2,3,4,5,6-Pentachloro-1,1'-biphenyl |
| PCB117 | C1=CC(=CC=C1C2=C(C(=CC(=C2Cl)Cl)Cl)Cl)Cl | 2,3,4',5,6-Pentachloro-1,1'-biphenyl |
| PCB118 | C1=CC(=C(C=C1C2=CC(=C(C=C2Cl)Cl)Cl)Cl)Cl | 2,3',4,4',5-Pentachloro-1,1'-biphenyl |
| PCB119 | C1=CC(=C(C=C1C2=C(C=C(C=C2Cl)Cl)Cl)Cl)Cl | 2,3',4,4',6-Pentachloro-1,1'-biphenyl |
| PCB120 | C1=C(C=C(C=C1Cl)Cl)C2=CC(=C(C=C2Cl)Cl)Cl | 2,3',4,5,5'-Pentachloro-1,1'-biphenyl |
| PCB121 | C1=C(C=C(C=C1Cl)Cl)C2=C(C=C(C=C2Cl)Cl)Cl | 2,3',4,5',6-Pentachloro-1,1'-biphenyl |
| PCB122 | C1=CC(=C(C(=C1)Cl)Cl)C2=CC(=C(C(=C2)Cl)Cl)Cl | 2,3,3',4',5'-Pentachloro-1,1'-biphenyl |
| PCB123 | C1=CC(=C(C=C1Cl)Cl)C2=CC(=C(C(=C2)Cl)Cl)Cl | 2,3',4,4',5'-Pentachloro-1,1'-biphenyl |
| PCB124 | C1=CC(=C(C=C1Cl)C2=CC(=C(C(=C2)Cl)Cl)Cl)Cl | 2,3',4',5,5'-Pentachloro-1,1'-biphenyl |
| PCB125 | C1=CC(=C(C(=C1)Cl)C2=CC(=C(C(=C2)Cl)Cl)Cl)Cl | 2,3',4',5',6-Pentachloro-1,1'-biphenyl |
| PCB126 | C1=CC(=C(C=C1C2=CC(=C(C(=C2)Cl)Cl)Cl)Cl)Cl | 3,3',4,4',5-Pentachloro-1,1'-biphenyl |
| PCB127 | C1=C(C=C(C=C1Cl)Cl)C2=CC(=C(C(=C2)Cl)Cl)Cl | 3,3',4,5,5'-Pentachloro-1,1'-biphenyl |
| PCB128 | C1=CC(=C(C(=C1C2=C(C(=C(C=C2)Cl)Cl)Cl)Cl)Cl)Cl | 2,2',3,3',4,4'-Hexachloro-1,1'-biphenyl |
| PCB129 | C1=CC(=C(C(=C1)Cl)Cl)C2=CC(=C(C(=C2Cl)Cl)Cl)Cl | 2,2',3,3',4,5-Hexachloro-1,1'-biphenyl |
| PCB130 | C1=CC(=C(C(=C1C2=C(C(=CC(=C2)Cl)Cl)Cl)Cl)Cl)Cl | 2,2',3,3',4,5'-Hexachloro-1,1'-biphenyl |
| PCB131 | C1=CC(=C(C(=C1)Cl)Cl)C2=C(C(=C(C=C2Cl)Cl)Cl)Cl | 2,2',3,3',4,6-Hexachloro-1,1'-biphenyl |
| PCB132 | C1=CC(=C(C(=C1C2=C(C=CC(=C2Cl)Cl)Cl)Cl)Cl)Cl | 2,2',3,3',4,6'-Hexachloro-1,1'-biphenyl |
| PCB133 | C1=C(C=C(C(=C1C2=C(C(=CC(=C2)Cl)Cl)Cl)Cl)Cl)Cl | 2,2',3,3',5,5'-Hexachloro-1,1'-biphenyl |
| PCB134 | C1=CC(=C(C(=C1)Cl)Cl)C2=C(C(=CC(=C2Cl)Cl)Cl)Cl | 2,2',3,3',5,6-Hexachloro-1,1'-biphenyl |
| PCB135 | C1=CC(=C(C(=C1Cl)C2=C(C(=CC(=C2)Cl)Cl)Cl)Cl)Cl | 2,2',3,3',5,6'-Hexachloro-1,1'-biphenyl |
| PCB136 | C1=CC(=C(C(=C1Cl)C2=C(C=CC(=C2Cl)Cl)Cl)Cl)Cl | 2,2',3,3',6,6'-Hexachloro-1,1'-biphenyl |
| PCB137 | C1=CC(=C(C=C1Cl)Cl)C2=CC(=C(C(=C2Cl)Cl)Cl)Cl | 2,2',3,4,4',5-Hexachloro-1,1'-biphenyl |
| PCB138 | C1=CC(=C(C(=C1C2=CC(=C(C=C2Cl)Cl)Cl)Cl)Cl)Cl | 2,2',3,4,4',5'-Hexachloro-1,1'-biphenyl |
| PCB139 | C1=CC(=C(C=C1Cl)Cl)C2=C(C(=C(C=C2Cl)Cl)Cl)Cl | 2,2',3,4,4',6-Hexachloro-1,1'-biphenyl |
| PCB140 | C1=CC(=C(C(=C1C2=C(C=C(C=C2Cl)Cl)Cl)Cl)Cl)Cl | 2,2',3,4,4',6'-Hexachloro-1,1'-biphenyl |
| PCB141 | C1=CC(=C(C=C1Cl)C2=CC(=C(C(=C2Cl)Cl)Cl)Cl)Cl | 2,2',3,4,5,5'-Hexachloro-1,1'-biphenyl |
| PCB142 | C1=CC=C(C(=C1)C2=C(C(=C(C(=C2Cl)Cl)Cl)Cl)Cl)Cl | 2,2',3,4,5,6-Hexachloro-1,1'-biphenyl |
| PCB143 | C1=CC(=C(C(=C1)Cl)C2=CC(=C(C(=C2Cl)Cl)Cl)Cl)Cl | 2,2',3,4,5,6'-Hexachloro-1,1'-biphenyl |
| PCB144 | C1=CC(=C(C=C1Cl)C2=C(C(=C(C=C2Cl)Cl)Cl)Cl)Cl | 2,2',3,4,5',6-Hexachloro-1,1'-biphenyl |
| PCB145 | C1=CC(=C(C(=C1)Cl)C2=C(C(=C(C=C2Cl)Cl)Cl)Cl)Cl | 2,2',3,4,6,6'-Hexachloro-1,1'-biphenyl |
| PCB146 | C1=C(C=C(C(=C1C2=CC(=C(C=C2Cl)Cl)Cl)Cl)Cl)Cl | 2,2',3,4',5,5'-Hexachloro-1,1'-biphenyl |
| PCB147 | C1=CC(=C(C=C1Cl)Cl)C2=C(C(=CC(=C2Cl)Cl)Cl)Cl | 2,2',3,4',5,6-Hexachloro-1,1'-biphenyl |
| PCB148 | C1=C(C=C(C(=C1C2=C(C=C(C=C2Cl)Cl)Cl)Cl)Cl)Cl | 2,2',3,4',5,6'-Hexachloro-1,1'-biphenyl |
| PCB149 | C1=CC(=C(C(=C1Cl)C2=CC(=C(C=C2Cl)Cl)Cl)Cl)Cl | 2,2',3,4',5',6-Hexachloro-1,1'-biphenyl |
| PCB150 | C1=CC(=C(C(=C1Cl)C2=C(C=C(C=C2Cl)Cl)Cl)Cl)Cl | 2,2',3,4',6,6'-Hexachloro-1,1'-biphenyl |
| PCB151 | C1=CC(=C(C=C1Cl)C2=C(C(=CC(=C2Cl)Cl)Cl)Cl)Cl | 2,2',3,5,5',6-Hexachloro-1,1'-biphenyl |
| PCB152 | C1=CC(=C(C(=C1)Cl)C2=C(C(=CC(=C2Cl)Cl)Cl)Cl)Cl | 2,2',3,5,6,6'-Hexachloro-1,1'-biphenyl |
| PCB153 | C1=C(C(=CC(=C1Cl)Cl)Cl)C2=CC(=C(C=C2Cl)Cl)Cl | 2,2',4,4',5,5'-Hexachloro-1,1'-biphenyl |
| PCB154 | C1=C(C=C(C(=C1Cl)C2=CC(=C(C=C2Cl)Cl)Cl)Cl)Cl | 2,2',4,4',5,6'-Hexachloro-1,1'-biphenyl |
| PCB155 | C1=C(C=C(C(=C1Cl)C2=C(C=C(C=C2Cl)Cl)Cl)Cl)Cl | 2,2',4,4',6,6'-Hexachloro-1,1'-biphenyl |
| PCB156 | C1=CC(=C(C=C1C2=CC(=C(C(=C2Cl)Cl)Cl)Cl)Cl)Cl | 2,3,3',4,4',5-Hexachloro-1,1'-biphenyl |
| PCB157 | C1=CC(=C(C(=C1C2=CC(=C(C(=C2)Cl)Cl)Cl)Cl)Cl)Cl | 2,3,3',4,4',5'-Hexachloro-1,1'-biphenyl |
| PCB158 | C1=CC(=C(C=C1C2=C(C(=C(C=C2Cl)Cl)Cl)Cl)Cl)Cl | 2,3,3',4,4',6-Hexachloro-1,1'-biphenyl |
| PCB159 | C1=C(C=C(C=C1Cl)Cl)C2=CC(=C(C(=C2Cl)Cl)Cl)Cl | 2,3,3',4,5,5'-Hexachloro-1,1'-biphenyl |
| PCB160 | C1=CC(=CC(=C1)Cl)C2=C(C(=C(C(=C2Cl)Cl)Cl)Cl)Cl | 2,3,3',4,5,6-Hexachloro-1,1'-biphenyl |
| PCB161 | C1=C(C=C(C=C1Cl)Cl)C2=C(C(=C(C=C2Cl)Cl)Cl)Cl | 2,3,3',4,5',6-Hexachloro-1,1'-biphenyl |
| PCB162 | C1=C(C=C(C(=C1Cl)Cl)Cl)C2=C(C(=CC(=C2)Cl)Cl)Cl | 2,3,3',4',5,5'-Hexachloro-1,1'-biphenyl |
| PCB163 | C1=CC(=C(C=C1C2=C(C(=CC(=C2Cl)Cl)Cl)Cl)Cl)Cl | 2,3,3',4',5,6-Hexachloro-1,1'-biphenyl |
| PCB164 | C1=CC(=C(C(=C1Cl)C2=CC(=C(C(=C2)Cl)Cl)Cl)Cl)Cl | 2,3,3',4',5',6-Hexachloro-1,1'-biphenyl |
| PCB165 | C1=C(C=C(C=C1Cl)Cl)C2=C(C(=CC(=C2Cl)Cl)Cl)Cl | 2,3,3',5,5',6-Hexachloro-1,1'-biphenyl |
| PCB166 | C1=CC(=CC=C1C2=C(C(=C(C(=C2Cl)Cl)Cl)Cl)Cl)Cl | 2,3,4,4',5,6-Hexachloro-1,1'-biphenyl |
| PCB167 | C1=C(C=C(C(=C1Cl)Cl)Cl)C2=CC(=C(C=C2Cl)Cl)Cl | 2,3',4,4',5,5'-Hexachloro-1,1'-biphenyl |
| PCB168 | C1=C(C=C(C(=C1Cl)Cl)Cl)C2=C(C=C(C=C2Cl)Cl)Cl | 2,3',4,4',5',6-Hexachloro-1,1'-biphenyl |
| PCB169 | C1=C(C=C(C(=C1Cl)Cl)Cl)C2=CC(=C(C(=C2)Cl)Cl)Cl | 3,3',4,4',5,5'-Hexachloro-1,1'-biphenyl |
| PCB170 | C1=CC(=C(C(=C1C2=CC(=C(C(=C2Cl)Cl)Cl)Cl)Cl)Cl)Cl | 2,2',3,3',4,4',5-Heptachloro-1,1'-biphenyl |
| PCB171 | C1=CC(=C(C(=C1C2=C(C(=C(C=C2Cl)Cl)Cl)Cl)Cl)Cl)Cl | 2,2',3,3',4,4',6-Heptachloro-1,1'-biphenyl |
| PCB172 | C1=C(C=C(C(=C1C2=CC(=C(C(=C2Cl)Cl)Cl)Cl)Cl)Cl)Cl | 2,2',3,3',4,5,5'-Heptachloro-1,1'-biphenyl |
| PCB173 | C1=CC(=C(C(=C1)Cl)Cl)C2=C(C(=C(C(=C2Cl)Cl)Cl)Cl)Cl | 2,2',3,3',4,5,6-Heptachloro-1,1'-biphenyl |
| PCB174 | C1=CC(=C(C(=C1Cl)C2=CC(=C(C(=C2Cl)Cl)Cl)Cl)Cl)Cl | 2,2',3,3',4,5,6'-Heptachloro-1,1'-biphenyl |
| PCB175 | C1=C(C=C(C(=C1C2=C(C(=C(C=C2Cl)Cl)Cl)Cl)Cl)Cl)Cl | 2,2',3,3',4,5',6-Heptachloro-1,1'-biphenyl |
| PCB176 | C1=CC(=C(C(=C1Cl)C2=C(C(=C(C=C2Cl)Cl)Cl)Cl)Cl)Cl | 2,2',3,3',4,6,6'-Heptachloro-1,1'-biphenyl |
| PCB177 | C1=CC(=C(C(=C1C2=C(C(=CC(=C2Cl)Cl)Cl)Cl)Cl)Cl)Cl | 2,2',3,3',4,5',6'-Heptachloro-1,1'-biphenyl |
| PCB178 | C1=C(C=C(C(=C1C2=C(C(=CC(=C2Cl)Cl)Cl)Cl)Cl)Cl)Cl | 2,2',3,3',5,5',6-Heptachloro-1,1'-biphenyl |
| PCB179 | C1=CC(=C(C(=C1Cl)C2=C(C(=CC(=C2Cl)Cl)Cl)Cl)Cl)Cl | 2,2',3,3',5,6,6'-Heptachloro-1,1'-biphenyl |
| PCB180 | C1=C(C(=CC(=C1Cl)Cl)Cl)C2=CC(=C(C(=C2Cl)Cl)Cl)Cl | 2,2',3,4,4',5,5'-Heptachloro-1,1'-biphenyl |
| PCB181 | C1=CC(=C(C=C1Cl)Cl)C2=C(C(=C(C(=C2Cl)Cl)Cl)Cl)Cl | 2,2',3,4,4',5,6-Heptachloro-1,1'-biphenyl |
| PCB182 | C1=C(C=C(C(=C1Cl)C2=CC(=C(C(=C2Cl)Cl)Cl)Cl)Cl)Cl | 2,2',3,4,4',5,6'-Heptachloro-1,1'-biphenyl |
| PCB183 | C1=C(C(=CC(=C1Cl)Cl)Cl)C2=C(C(=C(C=C2Cl)Cl)Cl)Cl | 2,2',3,4,4',5',6-Heptachloro-1,1'-biphenyl |
| PCB184 | C1=C(C=C(C(=C1Cl)C2=C(C(=C(C=C2Cl)Cl)Cl)Cl)Cl)Cl | 2,2',3,4,4',6,6'-Heptachloro-1,1'-biphenyl |
| PCB185 | C1=CC(=C(C=C1Cl)C2=C(C(=C(C(=C2Cl)Cl)Cl)Cl)Cl)Cl | 2,2',3,4,5,5',6-Heptachloro-1,1'-biphenyl |
| PCB186 | C1=CC(=C(C(=C1)Cl)C2=C(C(=C(C(=C2Cl)Cl)Cl)Cl)Cl)Cl | 2,2',3,4,5,6,6'-Heptachloro-1,1'-biphenyl |
| PCB187 | C1=C(C(=CC(=C1Cl)Cl)Cl)C2=C(C(=CC(=C2Cl)Cl)Cl)Cl | 2,2',3,4',5,5',6-Heptachloro-1,1'-biphenyl |
| PCB188 | C1=C(C=C(C(=C1Cl)C2=C(C(=CC(=C2Cl)Cl)Cl)Cl)Cl)Cl | 2,2',3,4',5,6,6'-Heptachloro-1,1'-biphenyl |
| PCB189 | C1=C(C=C(C(=C1Cl)Cl)Cl)C2=CC(=C(C(=C2Cl)Cl)Cl)Cl | 2,3,3',4,4',5,5'-Heptachloro-1,1'-biphenyl |
| PCB190 | C1=CC(=C(C=C1C2=C(C(=C(C(=C2Cl)Cl)Cl)Cl)Cl)Cl)Cl | 2,3,3',4,4',5,6-Heptachloro-1,1'-biphenyl |
| PCB191 | C1=C(C=C(C(=C1Cl)Cl)Cl)C2=C(C(=C(C=C2Cl)Cl)Cl)Cl | 2,3,3',4,4',5',6-Heptachloro-1,1'-biphenyl |
| PCB192 | C1=C(C=C(C=C1Cl)Cl)C2=C(C(=C(C(=C2Cl)Cl)Cl)Cl)Cl | 2,3,3',4,5,5',6-Heptachloro-1,1'-biphenyl |
| PCB193 | C1=C(C=C(C(=C1Cl)Cl)Cl)C2=C(C(=CC(=C2Cl)Cl)Cl)Cl | 2,3,3',4',5,5',6-Heptachloro-1,1'-biphenyl |
| PCB194 | C1=C(C(=C(C(=C1Cl)Cl)Cl)Cl)C2=CC(=C(C(=C2Cl)Cl)Cl)Cl | 2,2',3,3',4,4',5,5'-Octachloro-1,1'-biphenyl |
| PCB195 | C1=CC(=C(C(=C1C2=C(C(=C(C(=C2Cl)Cl)Cl)Cl)Cl)Cl)Cl)Cl | 2,2',3,3',4,4',5,6-Octachloro-1,1'-biphenyl |
| PCB196 | C1=C(C(=C(C(=C1Cl)Cl)Cl)Cl)C2=C(C(=C(C=C2Cl)Cl)Cl)Cl | 2,2',3,3',4,4',5,6'-Octachloro-1,1'-biphenyl |
| PCB197 | C1=C(C(=C(C(=C1Cl)Cl)Cl)C2=C(C(=C(C=C2Cl)Cl)Cl)Cl)Cl | 2,2',3,3',4,4',6,6'-Octachloro-1,1'-biphenyl |
| PCB198 | C1=C(C=C(C(=C1C2=C(C(=C(C(=C2Cl)Cl)Cl)Cl)Cl)Cl)Cl)Cl | 2,2',3,3',4,5,5',6-Octachloro-1,1'-biphenyl |
| PCB199 | C1=C(C(=C(C(=C1Cl)Cl)Cl)Cl)C2=C(C(=CC(=C2Cl)Cl)Cl)Cl | 2,2',3,3',4,5,5',6'-Octachloro-1,1'-biphenyl |
| PCB200 | C1=CC(=C(C(=C1Cl)C2=C(C(=C(C(=C2Cl)Cl)Cl)Cl)Cl)Cl)Cl | 2,2',3,3',4,5,6,6'-Octachloro-1,1'-biphenyl |
| PCB201 | C1=C(C(=C(C(=C1Cl)Cl)Cl)C2=C(C(=CC(=C2Cl)Cl)Cl)Cl)Cl | 2,2',3,3',4,5',6,6'-Octachloro-1,1'-biphenyl |
| PCB202 | C1=C(C(=C(C(=C1Cl)Cl)C2=C(C(=CC(=C2Cl)Cl)Cl)Cl)Cl)Cl | 2,2',3,3',5,5',6,6'-Octachloro-1,1'-biphenyl |
| PCB203 | C1=C(C(=CC(=C1Cl)Cl)Cl)C2=C(C(=C(C(=C2Cl)Cl)Cl)Cl)Cl | 2,2',3,4,4',5,5',6-Octachloro-1,1'-biphenyl |
| PCB204 | C1=C(C=C(C(=C1Cl)C2=C(C(=C(C(=C2Cl)Cl)Cl)Cl)Cl)Cl)Cl | 2,2',3,4,4',5,6,6'-Octachloro-1,1'-biphenyl |
| PCB205 | C1=C(C=C(C(=C1Cl)Cl)Cl)C2=C(C(=C(C(=C2Cl)Cl)Cl)Cl)Cl | 2,3,3',4,4',5,5',6-Octachloro-1,1'-biphenyl |
| PCB206 | C1=C(C(=C(C(=C1Cl)Cl)Cl)Cl)C2=C(C(=C(C(=C2Cl)Cl)Cl)Cl)Cl | 2,2',3,3',4,4',5,5',6-Nonachloro-1,1'-biphenyl |
| PCB207 | C1=C(C(=C(C(=C1Cl)Cl)Cl)C2=C(C(=C(C(=C2Cl)Cl)Cl)Cl)Cl)Cl | 2,2',3,3',4,4',5,6,6'-Nonachloro-1,1'-biphenyl |
| PCB208 | C1=C(C(=C(C(=C1Cl)Cl)C2=C(C(=C(C(=C2Cl)Cl)Cl)Cl)Cl)Cl)Cl | 2,2',3,3',4,5,5',6,6'-Nonachloro-1,1'-biphenyl |
| PCB209 | C1(=C(C(=C(C(=C1Cl)Cl)Cl)Cl)Cl)C2=C(C(=C(C(=C2Cl)Cl)Cl)Cl)Cl | 2,2',3,3',4,4',5,5',6,6'-Decachloro-1,1'-biphenyl |

The table was generated from a link created by the Iowa Superfund Research Program (ISRP) and is accessible from https://apps.iowasuperfund.uiowa.edu/pcb-ids/.

**Table S2.** The canonical SMILES structure of the 124 available methoxylated PCBs used as calibration standards for corresponding OH-PCBs.

| **Compound ID** | **Compound name** | **Chlorine pattern** | **MeO pattern** | **Canonical SMILES** |
| --- | --- | --- | --- | --- |
| meopcb001 | 2-MeO-PCB3 | 4 | 2 | ClC(C=C1OC)=CC=C1C2=CC=CC=C2 |
| meopcb002 | 2'-MeO-PCB3 | 4 | 2' | ClC(C=C1)=CC=C1C2=C(OC)C=CC=C2 |
| meopcb003 | 3-MeO-PCB3 | 4 | 3 | ClC(C(OC)=C1)=CC=C1C2=CC=CC=C2 |
| meopcb004 | 2-MeO-PCB11 | 3,3' | 2 | ClC1=C(OC)C(C2=CC(Cl)=CC=C2)=CC=C1 |
| meopcb005 | 4,5-diMeO-PCB1 | 2 | 4,5 | ClC1=CC(OC)=C(OC)C=C1C2=CC=CC=C2 |
| meopcb006 | 2',3'-diMeO-PCB3 | 4 | 2',3' | ClC(C=C1)=CC=C1C2=C(OC)C(OC)=CC=C2 |
| meopcb007 | 6-MeO-PCB11 | 3,3' | 6 | ClC1=CC=C(OC)C(C2=CC=CC(Cl)=C2)=C1 |
| meopcb008 | 2',3'-diMeO-PCB5 | 2,3 | 2',3' | ClC1=C(Cl)C(C2=C(OC)C(OC)=CC=C2)=CC=C1 |
| meopcb009 | 4'-MeO-PCB5 | 2,3 | 4' | ClC1=C(Cl)C(C2=CC=C(OC)C=C2)=CC=C1 |
| meopcb010 | 2'-MeO-PCB28 | 2,4,4' | 2' | ClC1=CC(Cl)=CC=C1C2=C(OC)C=C(Cl)C=C2 |
| meopcb011 | 2',5'-diMeO-PCB8 | 2,4' | 2'5' | ClC1=CC=CC=C1C2=C(OC)C=C(Cl)C(OC)=C2 |
| meopcb012 | 4,5-diMeO-PCB4 | 2,2' | 4,5 | ClC1=CC(OC)=C(OC)C=C1C2=C(Cl)C=CC=C2 |
| meopcb013 | 5-MeO-PCB11 | 3,3' | 5 | ClC1=CC(OC)=CC(C2=CC=CC(Cl)=C2)=C1 |
| meopcb014 | 3-MeO-PCB50 | 2,2',4,6 | 3 | ClC(C(OC)=C1Cl)=CC(Cl)=C1C2=C(Cl)C=CC=C2 |
| meopcb015 | 2',5'-diMeO-PCB5 | 2,3 | 2',5' | ClC1=C(Cl)C=CC=C1C2=C(OC)C=CC(OC)=C2 |
| meopcb016 | 3',4'-diMeO-PCB3 | 4 | 3',4' | ClC(C=C1)=CC=C1C2=CC=C(OC)C(OC)=C2 |
| meopcb017 | 5,6-diMeO-PCB11 | 3,3' | 5,6 | ClC1=CC(OC)=C(OC)C(C2=CC=CC(Cl)=C2)=C1 |
| meopcb018 | 2,5-diMeO-PCB11 | 3,3' | 2,5 | ClC1=CC(OC)=CC(C2=CC=CC(Cl)=C2)=C1OC |
| meopcb019 | 4-MeO-PCB11 | 3,3' | 4 | ClC1=C(OC)C=CC(C2=CC=CC(Cl)=C2)=C1 |
| meopcb020 | 3-MeO-PCB28 | 2,4,4' | 3 | ClC1=C(OC)C(Cl)=C(C2=CC=C(Cl)C=C2)C=C1 |
| meopcb021 | 2',3'-diMeO-PCB12 | 3,4 | 2',3' | ClC(C(Cl)=C1)=CC=C1C2=C(OC)C(OC)=CC=C2 |
| meopcb022 | 2',3'-diMeO-PCB9 | 2,5 | 2',3' | ClC1=CC(C2=C(OC)C(OC)=CC=C2)=C(Cl)C=C1 |
| meopcb023 | 4'-MeO-PCB12 | 3,4 | 4' | ClC1=CC(C2=CC=C(OC)C=C2)=CC=C1Cl |
| meopcb024 | 4'-MeO-PCB35 | 3,3',4 | 4' | ClC1=CC(C2=CC(Cl)=C(Cl)C=C2)=CC=C1OC |
| meopcb025 | 2',5'-MeO-PCB14 | 3,5 | 2,5' | ClC1=CC(C2=CC(OC)=CC=C2OC)=CC(Cl)=C1 |
| meopcb026 | 3'-MeO-PCB28 | 2,4,4' | 3' | ClC1=CC(Cl)=C(C2=CC=C(Cl)C(OC)=C2)C=C1 |
| meopcb027 | 5-MeO-PCB28 | 2,4,4' | 5 | ClC1=CC(Cl)=C(C2=CC=C(Cl)C=C2)C=C1OC |
| meopcb028 | 3',4'-diMeO-PCB5 | 2,3 | 3',4' | ClC1=C(Cl)C=CC=C1C2=CC=C(OC)C(OC)=C2 |
| meopcb029 | 4,5-diMeO-PCB8 | 2,4' | 4,5 | ClC1=CC(OC)=C(OC)C=C1C2=CC=C(Cl)C=C2 |
| meopcb030 | 2,5-diMeO-PCB15 | 4,4' | 2,5 | ClC(C=C1)=CC=C1C2=CC(OC)=C(Cl)C=C2OC |
| meopcb031 | 4'-MeO-PCB29 | 2,4,5 | 4' | ClC1=C(Cl)C=C(C2=CC=C(OC)C=C2)C(Cl)=C1 |
| meopcb032 | 4-MeO-PCB36 | 3,3',5 | 4 | ClC1=C(OC)C(Cl)=CC(C2=CC=CC(Cl)=C2)=C1 |
| meopcb033 | 4'-MeO-PCB25 | 2,3'4 | 4' | ClC1=CC(Cl)=C(C2=CC=C(OC)C(Cl)=C2)C=C1 |
| meopcb034 | 4,5-diMeO-PCB11 | 2,4' | 4,5 | ClC1=C(OC)C(OC)=CC(C2=CC=CC(Cl)=C2)=C1 |
| meopcb035 | 2',5'-diMeO-PCB31 | 2,4',5 | 2',5' | ClC(C(OC)=C1)=CC(OC)=C1C2=C(Cl)C=CC(Cl)=C2 |
| meopcb036 | 3-MeO-PCB103 | 2,2',4,5',6 | 3 | ClC1=CC(C2=C(Cl)C(OC)=C(Cl)C=C2Cl)=C(Cl)C=C1 |
| meopcb037 | 3',4'-diMeO-PCB14 | 3,5 | 3',4' | ClC1=CC(C2=CC(OC)=C(OC)C=C2)=CC(Cl)=C1 |
| meopcb038 | 3-MeO-PCB100 | 2,2',4,4',6 | 3 | ClC1=C(C2=C(Cl)C=C(Cl)C=C2)C(Cl)=CC(Cl)=C1OC |
| meopcb039 | 4'-MeO-PCB68 | 2,3',4,5' | 4' | ClC1=C(C2=CC(Cl)=C(OC)C(Cl)=C2)C=CC(Cl)=C1 |
| meopcb040 | 3'-MeO-PCB98 | 2,2',3,4',6' | 3' | ClC1=C(C2=C(Cl)C=C(Cl)C(OC)=C2Cl)C=CC=C1Cl |
| meopcb041 | 3',4'-diMeO-PCB12 | 3,4 | 3',4' | ClC1=CC=C(C2=CC(OC)=C(OC)C=C2)C=C1Cl |
| meopcb042 | 3'-MeO-PCB150 | 2,2',3,4',6,6' | 3' | ClC1=C(C2=C(Cl)C=C(Cl)C(OC)=C2Cl)C(Cl)=CC=C1Cl |
| meopcb043 | 4'-MeO-PCB95 | 2,2',3,5',6 | 4' | ClC1=C(C2=CC(Cl)=C(OC)C=C2Cl)C(Cl)=CC=C1Cl |
| meopcb044 | 5-MeO-PCB91 | 2,2',3,4',6 | 5 | ClC1=C(C2=CC=C(Cl)C=C2Cl)C(Cl)=C(OC)C=C1Cl |
| meopcb045 | 4,5-diMeO-PCB95 | 2,2',3,5',6 | 4,5 | ClC1=C(C2=CC(Cl)=CC=C2Cl)C(Cl)=C(OC)C(OC)=C1Cl |
| meopcb046 | 4-MeO-PCB91 | 2,2',3,4',6 | 4 | ClC1=C(C2=CC=C(Cl)C=C2Cl)C(Cl)=CC(OC)=C1Cl |
| meopcb047 | 4,5-diMeO-PCB91 | 2,2',3,4',6 | 4,5 | ClC1=C(C2=CC=C(Cl)C=C2Cl)C(Cl)=C(OC)C(OC)=C1Cl |
| meopcb048 | 4,5-diMeO-PCB136 | 2,2',3,3',6,6' | 4,5 | ClC1=C(C2=C(Cl)C=CC(Cl)=C2Cl)C(Cl)=C(OC)C(OC)=C1Cl |
| meopcb049 | 4-MeO-PCB136 | 2,2',3,3',6,6' | 4 | ClC1=C(C2=C(Cl)C=CC(Cl)=C2Cl)C(Cl)=CC(OC)=C1Cl |
| meopcb050 | 3'-MeO-PCB140 | 2,2',3,4,4',6' | 3' | ClC1=C(C2=C(Cl)C=C(Cl)C(OC)=C2Cl)C=CC(Cl)=C1Cl |
| meopcb051 | 4,4'-diMeO-PCB52 | 2,2',5,5' | 4,4' | ClC1=C(C2=CC(Cl)=C(OC)C=C2Cl)C=C(Cl)C(OC)=C1 |
| meopcb052 | 4,5-diMeO-PCB132 | 2,2',3,3',4,6' | 4,5 | ClC1=C(C2=C(Cl)C=C(Cl)C(Cl)=C2Cl)C=C(OC)C(OC)=C1Cl |
| meopcb053 | 2-MeO-PCB2 | 3 | 2 | ClC1=CC=CC(C2=CC=CC=C2)=C1OC |
| meopcb054 | 2'-MeO-PCB2 | 3 | 2' | ClC1=CC=CC(C2=CC=CC=C2OC)=C1 |
| meopcb055 | 6-MeO-PCB2 | 3 | 6 | ClC1=CC=C(OC)C(C2=CC=CC=C2)=C1 |
| meopcb056 | 4-MeO-PCB1 | 2 | 4 | ClC1=CC(OC)=CC=C1C2=CC=CC=C2 |
| meopcb057 | 5-MeO-PCB2 | 3 | 5 | ClC1=CC(OC)=CC(C2=CC=CC=C2)=C1 |
| meopcb058 | 3'-MeO-PCB2 | 3 | 3' | ClC1=CC=CC(C2=CC=CC(OC)=C2)=C1 |
| meopcb059 | 2'-MeO-PCB5 | 2,3 | 2' | ClC1=C(C=CC=C1C2=C(C=CC=C2)OC)Cl |
| meopcb060 | 4-MeO-PCB2 | 3 | 4 | ClC1=C(OC)C=CC(C2=CC=CC=C2)=C1 |
| meopcb061 | 4'-MeO-PCB2 | 3 | 4' | ClC1=CC=CC(C2=CC=C(OC)C=C2)=C1 |
| meopcb062 | 4'-MeO-PCB3 | 4 | 4' | ClC1=CC=C(C2=CC=C(C=C2)OC)C=C1 |
| meopcb063 | 2'-MeO-PCB30 | 2,4,6 | 2' | ClC1=CC(Cl)=CC(Cl)=C1C2=CC=CC=C2OC |
| meopcb064 | 3'-MeO-PCB9 | 2,5 | 3' | ClC1=CC(C2=CC(OC)=CC=C2)=C(Cl)C=C1 |
| meopcb065 | 2'-MeO-PCB12 | 3,4 | 2' | ClC(C(Cl)=C1)=CC=C1C2=C(OC)C=CC=C2 |
| meopcb066 | 4-MeO-PCB14 | 3,5 | 4 | ClC1=CC(C2=CC=CC=C2)=CC(Cl)=C1OC |
| meopcb067 | 4'-MeO-PCB9 | 2,5 | 4' | ClC1=CC(C2=CC=C(OC)C=C2)=C(Cl)C=C1 |
| meopcb068 | 3'-MeO-PCB30 | 2,4,6 | 3' | ClC1=CC(Cl)=CC(Cl)=C1C2=CC=CC(OC)=C2 |
| meopcb069 | 6'-MeO-PCB26 | 2,3',5 | 6' | ClC1=C(C2=C(OC)C=CC(Cl)=C2)C=C(Cl)C=C1 |
| meopcb070 | 4'-MeO-PCB18 | 2,2',5 | 4' | ClC1=C(C2=CC=C(OC)C=C2Cl)C=C(Cl)C=C1 |
| meopcb071 | 4'-MeO-PCB30 | 2,4,6 | 4' | ClC1=CC(Cl)=CC(Cl)=C1C2=CC=C(OC)C=C2 |
| meopcb072 | 3-MeO-PCB54 | 2,2',6,6' | 3 | ClC1=C(C2=C(Cl)C=CC=C2Cl)C(Cl)=CC=C1OC |
| meopcb073 | 6'-MeO-PCB69 | 2,3',4,6 | 6' | ClC1=C(C2=C(OC)C=CC(Cl)=C2)C(Cl)=CC(Cl)=C1 |
| meopcb074 | 2'-MeO-PCB65 | 2,3,5,6 | 2' | ClC1=C(C2=CC=CC=C2OC)C(Cl)=C(Cl)C=C1Cl |
| meopcb075 | 4'-MeO-PCB26 | 2,3',5 | 4' | ClC1=C(C=C(C=C1)Cl)C2=CC=C(OC)C(Cl)=C2 |
| meopcb076 | 4-MeO-PCB65 | 2,3,5,6 | 4 | ClC1=C(C2=CC=CC=C2)C(Cl)=C(Cl)C(OC)=C1Cl |
| meopcb077 | 3'-MeO-PCB65 | 2,3,5,6 | 3' | ClC1=C(C2=CC=CC(OC)=C2)C(Cl)=C(Cl)C=C1Cl |
| meopcb078 | 2'-MeO-PCB61 | 2,3,4,5 | 2' | ClC1=C(C2=CC=CC=C2OC)C=C(Cl)C(Cl)=C1Cl |
| meopcb079 | 6'-MeO-PCB101 | 2,2',4,5,5' | 6' | ClC1=C(C2=C(OC)C(Cl)=CC=C2Cl)C=C(Cl)C(Cl)=C1 |
| meopcb080 | 4'-MeO-PCB72 | 2,3',5,5' | 4' | ClC1=C(C2=CC(Cl)=C(OC)C(Cl)=C2)C=C(Cl)C=C1 |
| meopcb081 | 4'-MeO-PCB69 | 2,3',4,6 | 4' | ClC1=C(C2=CC=C(OC)C(Cl)=C2)C(Cl)=CC(Cl)=C1 |
| meopcb082 | 4'-MeO-PCB65 | 2,3,5,6 | 4' | ClC1=C(C2=CC=C(OC)C=C2)C(Cl)=C(Cl)C=C1Cl |
| meopcb083 | 6'-MeO-PCB83 | 2,2',3,3',5 | 6' | ClC1=C(C2=C(OC)C=CC(Cl)=C2Cl)C=C(Cl)C=C1Cl |
| meopcb084 | 3'-MeO-PCB61 | 2,3,4,5 | 3' | ClC1=C(C2=CC=CC(OC)=C2)C=C(Cl)C(Cl)=C1Cl |
| meopcb085 | 4'-MeO-PCB93 | 2,2',3,5,6 | 4' | ClC1=C(C2=CC=C(OC)C=C2Cl)C(Cl)=C(Cl)C=C1Cl |
| meopcb086 | 4'-MeO-PCB61 | 2,3,4,5 | 4' | ClC1=C(C2=CC=C(OC)C=C2)C=C(Cl)C(Cl)=C1Cl |
| meopcb087 | 4'-MeO-PCB79 | 3,3',4,5' | 4' | ClC1=C(Cl)C=CC(C2=CC(Cl)=C(OC)C(Cl)=C2)=C1 |
| meopcb088 | 4'-MeO-PCB101 | 2,2',4,5,5' | 4' | ClC1=C(C2=CC(Cl)=C(OC)C=C2Cl)C=C(Cl)C(Cl)=C1 |
| meopcb089 | 2'-MeO-PCB106 | 2,3,3',4,5 | 2' | ClC1=C(Cl)C(Cl)=CC(C2=CC=CC(Cl)=C2OC)=C1Cl |
| meopcb090 | 2'-MeO-PCB114 | 2,3,4,4',5 | 2' | ClC1=C(Cl)C(Cl)=CC(C2=CC=C(Cl)C=C2OC)=C1Cl |
| meopcb091 | 3,3'di-MeO-PCB155 | 2,2',4,4',6,6' | 3,3' | ClC1=C(OC)C(Cl)=C(C2=C(Cl)C=C(Cl)C(OC)=C2Cl)C(Cl)=C1 |
| meopcb092 | 4'-MeO-PCB120 | 2,3',4,5,5' | 4' | ClC1=CC(Cl)=C(C2=CC(Cl)=C(OC)C(Cl)=C2)C=C1Cl |
| meopcb093 | 4-MeO-PCB134 | 2,2',3,3',5,6 | 4 | ClC1=C(C2=CC=CC(Cl)=C2Cl)C(Cl)=C(Cl)C(OC)=C1Cl |
| meopcb094 | 4'-MeO-PCB86 | 2,2',3,4,5 | 4' | ClC1=C(Cl)C(Cl)=C(C2=CC=C(OC)C=C2Cl)C=C1Cl |
| meopcb095 | 4-MeO-PCB97 | 2,2',3,4',5' | 4 | ClC1=C(C2=CC(Cl)=C(Cl)C=C2Cl)C=CC(OC)=C1Cl |
| meopcb096 | 4'-MeO-PCB108 | 2,3,3',4,5' | 4' | ClC1=C(Cl)C(Cl)=C(C2=CC(Cl)=C(OC)C(Cl)=C2)C=C1 |
| meopcb097 | 3-MeO-PCB118 | 2,3',4,4',5 | 3 | ClC1=C(OC)C(Cl)=C(C2=CC=C(Cl)C(Cl)=C2)C=C1Cl |
| meopcb098 | 4-MeO-PCB107 | 2,3,3',4',5 | 4 | ClC1=C(C2=CC=C(Cl)C(Cl)=C2)C=C(Cl)C(OC)=C1Cl |
| meopcb099 | 3'-MeO-PCB184 | 2,2',3,4,4',6,6' | 3' | ClC1=C(Cl)C(Cl)=C(C2=C(Cl)C=C(Cl)C(OC)=C2Cl)C(Cl)=C1 |
| meopcb100 | 4-MeO-PCB146 | 2,2',3,4',5,5' | 4 | ClC1=C(C2=CC(Cl)=C(Cl)C=C2Cl)C=C(Cl)C(OC)=C1Cl |
| meopcb101 | 3'-MeO-PCB138 | 2,2',3,4,4',5' | 3' | ClC1=C(Cl)C(Cl)=C(C2=CC(Cl)=C(Cl)C(OC)=C2Cl)C=C1 |
| meopcb102 | 4'-MeO-PCB130 | 2,2',3,3',4,5' | 4' | ClC1=C(Cl)C(Cl)=C(C2=CC(Cl)=C(OC)C(Cl)=C2Cl)C=C1 |
| meopcb103 | 4'-MeO-PCB127 | 3,3',4,5,5' | 4' | ClC1=C(Cl)C=C(C2=CC(Cl)=C(OC)C(Cl)=C2)C=C1Cl |
| meopcb104 | 4-MeO-PCB178 | 2,2',3,3',5,5',6 | 4 | ClC1=C(OC)C(Cl)=C(Cl)C(C2=CC(Cl)=CC(Cl)=C2Cl)=C1Cl |
| meopcb105 | 4-MeO-PCB163 | 2,3,3',4',5,6 | 4 | ClC1=C(OC)C(Cl)=C(Cl)C(C2=CC=C(Cl)C(Cl)=C2)=C1Cl |
| meopcb106 | 3'-MeO-PCB182 | 2,2',3,4,4',5,6' | 3' | ClC1=C(Cl)C(Cl)=C(C2=C(Cl)C=C(Cl)C(OC)=C2Cl)C=C1Cl |
| meopcb107 | 3'-MeO-PCB183 | 2,2',3,4,4',5',6 | 3' | ClC1=C(Cl)C(Cl)=C(C2=CC(Cl)=C(Cl)C(OC)=C2Cl)C(Cl)=C1 |
| meopcb108 | 5-MeO-PCB183 | 2,2',3,4,4',5',6 | 5 | ClC1=C(Cl)C(Cl)=C(C2=CC(Cl)=C(Cl)C=C2Cl)C(Cl)=C1OC |
| meopcb109 | 4-MeO-PCB187 | 2,2',3,4',5,5',6 | 4 | ClC1=C(OC)C(Cl)=C(Cl)C(C2=CC(Cl)=C(Cl)C=C2Cl)=C1Cl |
| meopcb110 | 5-MeO-PCB138 | 2,2',3,4,4',5' | 5 | ClC1=C(Cl)C(Cl)=C(C2=CC(Cl)=C(Cl)C=C2Cl)C=C1OC |
| meopcb111 | 4-MeO-PCB202 | 2,2',3,3',5,5',6,6' | 4 | ClC1=C(OC)C(Cl)=C(Cl)C(C2=C(Cl)C(Cl)=CC(Cl)=C2Cl)=C1Cl |
| meopcb112 | 4'-MeO-PCB177 | 2,2',3,3',4,5',6' | 4' | ClC1=C(Cl)C(Cl)=C(C2=C(Cl)C(Cl)=C(OC)C(Cl)=C2Cl)C=C1 |
| meopcb113 | 4'-MeO-PCB159 | 2,3',3',4,5,5' | 4' | ClC1=C(Cl)C(Cl)=C(C2=CC(Cl)=C(OC)C(Cl)=C2)C=C1Cl |
| meopcb114 | 4-MeO-PCB162 | 2,3,3',4',5,5' | 4 | ClC1=C(OC)C(Cl)=CC(C2=CC(Cl)=C(Cl)C(Cl)=C2)=C1Cl |
| meopcb115 | 4'-MeO-PCB201 | 2,2',3,3',4,5',6,6' | 4' | ClC1=C(Cl)C(Cl)=C(C2=C(Cl)C(Cl)=C(OC)C(Cl)=C2Cl)C(Cl)=C1 |
| meopcb116 | 4-MeO-PCB193 | 2,3,3',4',5,5',6 | 4 | ClC1=C(OC)C(Cl)=C(Cl)C(C2=CC(Cl)=C(Cl)C(Cl)=C2)=C1Cl |
| meopcb117 | 3'-MeO-PCB180 | 2,2'3,4,4',5,5' | 3' | ClC1=C(Cl)C(Cl)=C(C2=CC(Cl)=C(Cl)C(OC)=C2Cl)C=C1Cl |
| meopcb118 | 4'-MeO-PCB172 | 2,2',3,3',4,5,5' | 4' | ClC1=C(Cl)C(Cl)=C(C2=CC(Cl)=C(OC)C(Cl)=C2Cl)C=C1Cl |
| meopcb119 | 4,4'di-MeO-PCB202 | 2,2',3,3',5,5',6,6' | 4,4' | ClC1=C(OC)C(Cl)=C(Cl)C(C2=C(Cl)C(Cl)=C(OC)C(Cl)=C2Cl)=C1Cl |
| meopcb120 | 4'-MeO-PCB198 | 2,2',3,3',4,5,5',6 | 4' | ClC1=C(Cl)C(Cl)=C(C2=CC(Cl)=C(OC)C(Cl)=C2Cl)C(Cl)=C1Cl |
| meopcb121 | 4'-MeO-PCB200 | 2,2',3,3',4,5,6,6' | 4' | ClC1=C(Cl)C(Cl)=C(C2=C(Cl)C=C(OC)C(Cl)=C2Cl)C(Cl)=C1Cl |
| meopcb122 | 3'-MeO-PCB203 | 2,2',3,4,4',5,5',6 | 3' | ClC1=C(Cl)C(Cl)=C(C2=CC(Cl)=C(Cl)C(OC)=C2Cl)C(Cl)=C1Cl |
| meopcb123 | 4'-MeO-PCB199 | 2,2',3,3',4,5,5',6' | 4' | ClC1=C(Cl)C(Cl)=C(C2=C(Cl)C(Cl)=C(OC)C(Cl)=C2Cl)C=C1Cl |
| meopcb124 | 4'-MeO-PCB208 | 2,2',3,3',4,5,5',6,6' | 4' | ClC1=C(Cl)C(Cl)=C(C2=C(Cl)C(Cl)=C(OC)C(Cl)=C2Cl)C(Cl)=C1Cl |

**Table S3.** Body mass (g) of dams used in this study is unaltered by PCBs.

| **Body Mass (g)** | **PCB (mg/kg/d)** | | | |
| --- | --- | --- | --- | --- |
|  | **0** | **0.1** | **1** | **6** |
| n | 11 | 13 | 12 | 12 |
| Mean | 25.68 | 25.55 | 25.09 | 25.54 |
| Std. Deviation | 2.18 | 1.66 | 1.73 | 2.31 |
| F (3, 44) = 0.2009; p=0.8952 | | | | |

**Table S4.** Precursor ions, product ions, and collision energies for each analyte in the GC-MS/MS analysis.

| **Analyte** | **Precursor Ion (*m/z*)** | **Product Ion (*m/z*)** | **Collision Energy (eV)** |
| --- | --- | --- | --- |
| 3-F,4'-OH-PCB3 | 237 | 194 | 25 |
| 4'-OH-PCB9 | 252 | 209 | 20 |
| PCB11 | 222 | 152 | 25 |
| 2-OH-PCB11 | 252 | 202 | 25 |
| 4-OH-PCB11 | 252 | 209 | 25 |
| 5-OH-PCB11 | 252 | 222 | 20 |
| 6-OH-PCB11 | 252 | 202 | 25 |
| 5,6-diOH-PCB11 | 282 | 232 | 25 |
| 2,5-diOH-PCB11 | 282 | 232 | 25 |
| 4,5-diOH-PCB11 | 282 | 204 | 25 |
| PCB15 | 222 | 152 | 25 |
| PCB28 | 256 | 186 | 25 |
| d-PCB30 | 261 | 191 | 30 |
| 2'-OH-PCB28 | 286 | 236 | 25 |
| 3-OH-PCB28 | 286 | 243 | 25 |
| 3'-OH-PCB28 | 286 | 243 | 25 |
| 5-OH-PCB28 | 286 | 243 | 25 |
| 4'-OH-PCB25 | 286 | 243 | 25 |
| PCB52 | 292 | 222 | 25 |
| 4-OH-PCB52 | 322 | 279 | 20 |
| 4,4'-diOH-PCB52 | 352 | 337 | 20 |
| PCB84 | 326 | 256 | 25 |
| 4-OH-PCB91 | 356 | 313 | 25 |
| PCB95 | 326 | 256 | 25 |
| 4-OH-PCB95 | 356 | 313 | 25 |
| 4'-OH-PCB95 | 356 | 313 | 25 |
| 5-OH-PCB95 | 356 | 313 | 25 |
| 4,5-diOH-PCB95 | 386 | 343 | 25 |
| 3-OH-PCB103 | 356 | 306 | 25 |
| PCB101 | 326 | 256 | 25 |
| 4'-OH-PCB101 | 356 | 313 | 25 |
| 6'-OH-PCB101 | 356 | 306 | 25 |
| PCB117 | 326 | 256 | 25 |
| PCB118 | 326 | 256 | 20 |
| 3-OH-PCB118 | 356 | 313 | 25 |
| PCB135 | 360 | 290 | 25 |
| PCB138 | 360 | 290 | 25 |
| 3'-OH-PCB138 | 390 | 347 | 25 |
| 5-OH-PCB138 | 390 | 347 | 25 |
| PCB149 | 360 | 290 | 25 |
| PCB153 | 360 | 290 | 25 |
| 3-OH-PCB153 | 390 | 347 | 25 |
| 4-OH-PCB146 | 390 | 347 | 25 |
| 4'-OH-PCB159 | 390 | 375 | 15 |
| PCB180 | 394 | 323 | 25 |
| 3'-OH-PCB180 | 424 | 381 | 25 |
| PCB204 | 430 | 358 | 25 |

**Table S5.** Ongoing Precision Recovery (OPR) for selected PCBs and OH-PCBs in method blanks and tissue matrices. Values are mean ± SD.

| **PCB/OH-PCB** | **Method blank N=11** | **Bladder**  **N=1** | **Blood**  **N=2** | **Liver**  **N=2** | **Adipose**  **N=2** | **Urine**  **N=2** |
| --- | --- | --- | --- | --- | --- | --- |
| PCB11 | 78 ± 21 | 95 | 96 ± 15 | 125 ± 0 | 98 ± 2 | 82 ± 7 |
| PCB15 | 80 ± 24 | 97 | 100 ± 22 | 140 ± 9 | 99 ± 4 | 91 ± 14 |
| PCB28 | 83 ± 20 | 128 | 122 ± 39 | 112 ± 13 | 95 ± 20 | 84 ± 0 |
| 4'-OH-PCB9 | 68 ± 16 | 75 | 29 ± 10 | 97 ± 8 | 64 ± 3 | 64 ± 5 |
| PCB52 | 87 ± 19 | 106 | 108 ± 27 | 113 ± 33 | 106 ± 15 | 60 ± 9 |
| 4-OH-PCB11 | 94 ± 32 | 113 | 43 ± 19 | 118 ± 13 | 96 ± 10 | 103 ± 7 |
| PCB95 | 94 ± 21 | 104 | 113 ± 45 | 114 ± 37 | 111 ± 8 | 56 ± 8 |
| PCB84 | 97 ± 23 | 102 | 108 ± 39 | 113 ± 32 | 114 ± 10 | 52 ± 7 |
| 4'-OH-PCB25 | 102 ± 28 | 118 | 48 ± 13 | 116 ± 10 | 99 ± 0 | 95 ± 3 |
| PCB101 | 101 ± 31 | 107 | 117 ± 42 | 128 ± 14 | 106 ± 13 | 66 ± 13 |
| 4-OH-PCB52 | 99 ± 28 | 111 | 50 ± 10 | 106 ± 4 | 108 ± 15 | 115 ± 23 |
| PCB117 | 98 ± 28 | 111 | 91 ± 20 | 103 ± 5 | 123 ± 18 | 86 ± 22 |
| PCB135 | 78 ± 12 | 108 | 93 ± 17 | 69 ± 7 | 86 ± 12 | 47 ± 10 |
| PCB149 | 81 ± 12 | 109 | 104 ± 16 | 74 ± 5 | 95 ± 16 | 52 ± 12 |
| 5-OH-PCB95 | 88 ± 24 | 107 | 56 ± 9 | 102 ± 12 | 102 ± 9 | 147 ± 18 |
| PCB118 | 94 ± 30 | 123 | 114 ± 36 | 111 ± 14 | 119 ± 15 | 123 ± 27 |
| 4-OH-PCB95 | 91 ± 24 | 107 | 75 ± 13 | 109 ± 29 | 107 ± 4 | 148 ± 12 |
| 4-OH-PCB91 | 100 ± 33 | 109 | 82 ± 28 | 129 ± 64 | 99 ± 11 | 157 ± 10 |
| PCB153 | 80 ± 10 | 120 | 104 ± 23 | 91 ± 36 | 87 ± 7 | 87 ± 18 |
| PCB138 | 84 ± 9 | 114 | 109 ± 27 | 111 ± 20 | 92 ± 10 | 97 ± 12 |
| 3-OH-PCB153 | 87 ± 20 | 102 | 104 ± 20 | 77 ± 24 | 87 ± 1 | 90 ± 1 |
| 4-OH-PCB146 | 87 ± 16 | 105 | 96 ± 37 | 86 ± 17 | 88 ± 0 | 92 ± 6 |
| PCB180 | 90 ± 12 | 128 | 108 ± 9 | 102 ± 9 | 94 ± 3 | 97 ± 1 |
| 4'-OH-PCB159 | 84 ± 18 | 98 | 101 ± 40 | 85 ± 11 | 100 ± 3 | 91 ± 2 |

**Table S6.** Recoveries (%) of surrogate standards for PCB and OH-PCB in different biological matrices. Values are mean ± SD.

| **Surrogate Standards** | **Bladder (n=14)** | **Blood (n=28)** | **Liver (n=28)** | **Adipose (n=28)** | **Intestine (n=28)** | **Urine (n=28)** |
| --- | --- | --- | --- | --- | --- | --- |
| PCB15 | 98 ± 8 | 80 ± 14 | 120 ± 19 | 111 ± 29 | 97 ± 35 | 96 ± 10 |
| 4'-OH-PCB9 | 73 ± 9 | 36 ± 10 | 70 ± 16 | 58 ± 8 | 65 ± 30 | 78 ± 7 |
| PCB117 | 105 ± 5 | 91 ± 22 | 98 ± 28 | 132 ± 26 | 135 ± 62 | 114 ± 16 |
| 4-OH-PCB91 | 106 ± 5 | 72 ± 16 | 97 ± 26 | 127 ± 31 | 111 ± 49 | 144 ± 18 |
| 4'-OH-PCB159 | 94 ± 5 | 101 ± 19 | 79 ± 18 | 102 ±14 | 81 ± 15 | 94 ± 7 |

**Table S7.** Method Detection Limits (MDLs) and Limits of Detection (LODs) for PCBs from MARBLES mixture and their possible OH-PCB metabolites.

| **PCB/OH-PCB** | **MDL^a^ (ng) (n=18)** | **LOD^b^ (ng/g)** | | | | |
| --- | --- | --- | --- | --- | --- | --- |
|  |  | **Bladder (n=8)** | **Blood (n=5)** | **Liver (n=5)** | **Adipose (n=5)** | **Urine (n=5)** |
| PCB11 | 0.10 | 6.42 | 0.47 | 0.48 | 1.64 | 1.41 |
| 2-OH-PCB11 | <0.01 | 0.01 | <0.01 | <0.01 | <0.01 | <0.01 |
| 4-OH-PCB11 | <0.01 | <0.01 | <0.01 | <0.01 | <0.01 | 2.95 |
| 5-OH-PCB11 | <0.01 | <0.01 | <0.01 | <0.01 | <0.01 | <0.01 |
| 6-OH-PCB11 | <0.01 | <0.01 | <0.01 | <0.01 | <0.01 | <0.01 |
| 5,6-OH-PCB11 | 0.01 | <0.01 | <0.01 | <0.01 | <0.01 | <0.01 |
| 2,5-OH-PCB11 | 0.01 | <0.01 | <0.01 | <0.01 | <0.01 | <0.01 |
| 4,5-OH-PCB11 | 0.05 | <0.01 | 0.28 | <0.01 | 0.42 | <0.01 |
| PCB28 | 0.12 | 6.34 | 0.63 | 2.81 | 18.1 | 6.58 |
| 2'-OH-PCB28 | 0.01 | 0.15 | 0.03 | <0.01 | 0.03 | <0.01 |
| 3-OH-PCB28 | 0.02 | <0.01 | 0.30 | <0.01 | <0.01 | 0.55 |
| 3'-OH-PCB28 | 0.02 | <0.01 | <0.01 | 0.52 | <0.01 | 5.85 |
| 5-OH-PCB28 | 0.05 | <0.01 | <0.01 | 0.92 | <0.01 | 5.25 |
| 4'-OH-PCB25^c^ | 0.07 | <0.01 | <0.01 | <0.01 | <0.01 | <0.01 |
| PCB52 | 0.05 | 2.14 | 0.25 | 0.20 | 1.82 | <0.01 |
| 4-OH-PCB52 | <0.01 | 1.49 | 1.83 | <0.01 | <0.01 | <0.01 |
| 4,4'-OH-PCB52 | <0.01 | <0.01 | <0.01 | <0.01 | <0.01 | <0.01 |
| PCB84 | 0.02 | 0.90 | 0.05 | 0.06 | <0.01 | <0.01 |
| PCB95 | 0.06 | 2.96 | 0.33 | 0.26 | 0.35 | 0.95 |
| 4-OH-PCB95 | <0.01 | <0.01 | <0.01 | <0.01 | <0.01 | <0.01 |
| 4'-OH-PCB95 | 0.02 | <0.01 | <0.01 | <0.01 | <0.01 | <0.01 |
| 5-OH-PCB95 | <0.01 | <0.01 | <0.01 | <0.01 | <0.01 | <0.01 |
| 4,5-OH-PCB95 | 0.02 | <0.01 | <0.01 | <0.01 | <0.01 | <0.01 |
| 3-OH-PCB103^c^ | 0.01 | 0.28 | 0.02 | <0.01 | <0.01 | <0.01 |
| PCB101 | 0.08 | 3.03 | 0.38 | 0.28 | 0.90 | 1.21 |
| 4'-OH-PCB101 | <0.01 | <0.01 | <0.01 | <0.01 | <0.01 | <0.01 |
| 6'-OH-PCB101 | <0.01 | <0.01 | <0.01 | <0.01 | <0.01 | <0.01 |
| PCB118 | 0.09 | 6.74 | 0.25 | 0.87 | 4.57 | 1.84 |
| 3-OH-PCB118 | <0.01 | <0.01 | <0.01 | <0.01 | <0.01 | <0.01 |
| PCB135 | 0.04 | 1.06 | <0.01 | 0.05 | 0.14 | <0.01 |
| PCB138 | 0.09 | 3.14 | 0.38 | 1.34 | 3.03 | 0.89 |
| 3'-OH-PCB138 | <0.01 | <0.01 | <0.01 | <0.01 | <0.01 | <0.01 |
| 5-OH-PCB138 | <0.01 | <0.01 | <0.01 | <0.01 | <0.01 | <0.01 |
| PCB149 | 0.30 | 1.30 | 0.82 | 0.10 | 0.41 | 0.39 |
| PCB153 | 0.25 | 8.22 | 1.07 | 0.90 | 7.34 | 1.72 |
| 3-OH-PCB153 | 0.03 | 0.44 | 0.07 | 0.16 | <0.01 | <0.01 |
| 4-OH-PCB146^c^ | 0.03 | 0.91 | 0.09 | 0.12 | <0.01 | <0.01 |
| PCB180 | 0.10 | 0.01 | <0.01 | 2.46 | 10.9 | <0.01 |
| 3'-OH-PCB180 | <0.01 | <0.01 | <0.01 | <0.01 | <0.01 | <0.01 |

^a^ MDL, Method Detection Limits (ng) were calculated using the formula: MDL= mean_blank_ + t_0.01, n-1_ *SD_blank_, where mean_blank_ is the mean of method blanks, t_0.01, n-1_ is Student’s t-value for n – 1 degrees of freedom at the 99% confidence level, and SD_blank_ is the standard deviation of the method blanks. N = 18

^b^ LOD, Limit of Detection (ng/g tissue weight) were adjusted by tissue mass and were calculated from formula: LOD= mean_control_ + t_0.01, n-1_ *SD_control_, where mean_control_ is the mean of control tissue measures, t_0.01, n-1_ is Student’s t-value for n – 1 degrees of freedom at the 99% confidence level, and SD_control_ is the standard deviation of the control tissue measures.

^c^ Possible NIH-shift products.

**Table S8.** Table of primers used for qPCR.

| **Gene** | **Tissue** | **Annealing Temperature (°C)/Notes on Primer concentration** | **Forward** | **Reverse** | **Potential additional products** |
| --- | --- | --- | --- | --- | --- |
| *Ppia* | Bladder | 60 | CATACAGGTCCTGGCATCTTG | TCATGCCTTCTTTCACCTTCC |  |
| *Cyp4x1* | Bladder | 58 | GAGCAACTCTGGCATTGGTTC | CTTGGCTGAGTTTCTCTCTACGG |  |
| *Cyp4x1* | Liver | 64 |  |  |  |
| *Cyp2s1* | Bladder | 66 | GGCCACCTCTAATGTCGTCTG | CCATGGAGAGCTGATCCCTAAC |  |
| *Cyp2s1* | Liver | 64 |  |  |  |
| *Ugt2b1* | Bladder | 61 | CCATGGCATTCCTATTGTTGG | TTCAAGGCAGTGAGAAGGTCTG |  |
| *Ugt2b1* | Liver | 60 |  |  |  |
| *Cyp1a1* | Bladder | 62; 1 μL primer | CAGCCTTCCCAAATGGTTTATG | ATGGACATGCAAGGACAGGAG | Transcript variant 1 and 2 |
| *Cyp1a1* | Liver | 60; 1 μL primer |  |  |  |
| *Cyp1a2* | Bladder | 62; 1 μL primer | CCAGCCCCTGCCCTTCAGTGGTA | TGGGAACCTGGGTCCTTGAGGC |  |
| *Cyp1a2* | Liver | 68 |  |  |  |
| *Cyp2b10* | Bladder | 62 | CCAAATCTCCAGGGCTCCAAGGC | TGCGGACTTGGGCTATTGGGAGG |  |
| *Cyp2b10* | Liver | 68 |  |  |  |
| *Cyp2b9* | Bladder | 60; 1 μL primer | CACCAGGACCCCATCCTCTA | TTTCTTGAAGCTGAATGAAACACT | *Cyp2b13 (1 base mismatch in each primer)* |
| *Cyp2b9* | Liver | 63 | CTGAGACCACAAGCGCCAC | CTTGACCATGAGCAGGACTCC |  |
| *Cyp3a11* | Bladder | 64 | ACAAGCAGGGATGGACCTGGTT | CCCATATCGGTAGAGGAGCACCA |  |
| *Cyp3a11* | Liver | 60; 1 μL primer |  |  |  |
| *Cyp3a41* | Bladder | 60 | GGCAGAAAGGTAGCCCTACAG | TGCCAGGAGGACCCATGTAT | *Cyp3a41a, Cyp3a41b; Cyp3a16 (1 base mismatch in each primer)* |
| *Cyp3a41* | Liver | 60 |  |  |  |
| *Cyp3a13* | Bladder | 62 | GTCATGGGTGAGTGGTTGCT | TCCCAGCTCCCAGGTTACAT |  |
| *Cyp3a16* | Bladder | 62 | TGCAGTCATAACTGGAGCGT | ACCAGGCATCAAAATCAATCAGT |  |
| *Cyp3a16* | Liver | 60 |  |  |  |
| *Pgk1* | Liver | 60 | TGGTGAAAGCCACTTCTAGGG | CATGGCTGACTTTATCCTCTGTG |  |
| Primer sequence and notes for each gene. Primers were diluted from a 100 μM stock to a 10 μM stock containing forward and reverse. Notes of 1 μL primer denote that 1 μL of this 10 μM forward and reverse stock was used in PCR reactions instead of 0.5 μL. | | | | | |

**Table S9**. The levels of PCB and OH-PCB (ng/g ww tissue) in bladder from female dam exposed via diet to 0.1, 1, or 6 mg/kg body weight of the MARBLES PCB mixture.

| **PCB/OH-PCB**  **names** | **0.1 mg/kg (n=4)** | | | **1 mg/kg (n=2)** | | | **6 mg/kg (n=3)** | | |
| --- | --- | --- | --- | --- | --- | --- | --- | --- | --- |
|  | **Mean** | **SD** | **f** | **Mean** | **SD** | **f** | **Mean** | **SD** | **f** |
| PCB11 | 7.35 |  | 1 | 18.3 |  | 1 | 72.9 | 83.0 | 3 |
| 2-OH-PCB11 |  |  |  |  |  |  |  |  |  |
| PCB28 | 202 | 190 | 4 | 1920 | 1740 | 2 | 10200 | 7910 | 3 |
| 6-OH-PCB11 |  |  |  |  |  |  | 0.07 | 0.07 | 2 |
| PCB52 | 12.6 | 10.1 | 4 | 108 | 96 | 2 | 560 | 417 | 3 |
| 2'-OH-PCB28 |  |  |  |  |  |  |  |  |  |
| 5-OH-PCB11 |  |  |  |  |  |  |  |  |  |
| 5,6-OH-PCB11 |  |  |  |  |  |  | 0.06 | 0.00 | 2 |
| 2,5-OH-PCB11 |  |  |  |  |  |  |  |  |  |
| 4-OH-PCB11 |  |  |  |  |  |  |  |  |  |
| 3-OH-PCB28 |  |  |  | 0.45 | 0.25 | 2 | 1.85 | 0.78 | 3 |
| PCB95 | 3.96 |  | 1 | 12.9 | 10.9 | 2 | 68.4 | 49.7 | 3 |
| PCB84 |  |  |  |  |  |  | 4.45 | 3.92 | 3 |
| 3'-OH-PCB28 | 0.16 | 0.19 | 3 | 0.83 | 0.02 | 2 | 6.76 | 4.54 | 3 |
| 5-OH-PCB28 |  |  |  | 0.34 | 0.01 | 2 | 2.29 | 1.42 | 3 |
| 4'-OH-PCB25 |  |  |  |  |  |  |  |  |  |
| PCB101 | 9.11 | 6.98 | 4 | 78.6 | 64.7 | 2 | 349 | 233 | 3 |
| 4,5-OH-PCB11 |  |  |  |  |  |  |  |  |  |
| 3-OH-PCB103 |  |  |  |  |  |  |  |  |  |
| 4-OH-PCB52 |  |  |  | 1.89 | 0.24 | 2 | 7.24 | 4.16 | 3 |
| 6'-OH-PCB101 |  |  |  |  |  |  |  |  |  |
| PCB135 | 2.10 |  | 1 | 8.34 | 7.18 | 2 | 35.3 | 24.6 | 3 |
| PCB149 | 2.67 | 1.65 | 3 | 19.0 | 14.9 | 2 | 79.5 | 50.5 | 3 |
| 5-OH-PCB95 |  |  |  |  |  |  |  |  |  |
| PCB118 | 65.4 | 94.8 | 4 | 1310 | 1080 | 2 | 6220 | 7630 | 3 |
| 4'-OH-PCB95 |  |  |  |  |  |  |  |  |  |
| 4-OH-PCB95 |  |  |  |  |  |  |  |  |  |
| 4,5-OH-PCB95 |  |  |  |  |  |  | 0.12 | 0.06 | 2 |
| PCB153 | 69.7 | 86.8 | 3 | 1120 | 848 | 2 | 5030 | 5840 | 3 |
| PCB138 | 27.5 | 41.9 | 4 | 563 | 513 | 2 | 2640 | 3280 | 3 |
| 4'-OH-PCB101 |  |  |  |  |  |  | 0.60 | 0.23 | 3 |
| 4,4'-OH-PCB52 |  |  |  |  |  |  |  |  |  |
| 3-OH-PCB118 | 0.10 | 0.06 | 4 | 0.96 | 0.02 | 2 | 5.14 | 3.64 | 3 |
| 3-OH-PCB153 | 0.75 |  | 1 | 0.96 | 0.13 | 2 | 5.29 | 4.56 | 3 |
| 4-OH-PCB146 |  |  |  |  |  |  |  |  |  |
| 3'-OH-PCB138 | 0.05 |  | 1 | 0.09 | 0.02 | 2 | 0.57 | 0.53 | 3 |
| PCB180 | 132 | 58 | 4 | 1030 | 646 | 2 | 5040 | 5520 | 3 |
| 5-OH-PCB138 |  |  |  |  |  |  |  |  |  |
| 3'-OH-PCB180 |  |  |  |  |  |  | 0.35 | 0.33 | 3 |
| **Total PCB** | 535 |  |  | 6185 |  |  | 30260 |  |  |
| **Total OH-PCB** | 1.06 |  |  | 5.52 |  |  | 30.3 |  |  |
| **Total** | 536 |  |  | 6190 |  |  | 30300 |  |  |

f: detection frequency. Empty boxes indicate values below LOD (limit of detection) or not available. Multiple bladders were pooled and used in analysis. Some of the pooled samples contain 1 tissue from a dam who failed to take care of their litter to weaning, but continued to be dosed to the wean date.

**Table S10**. The levels of PCB and OH-PCB (ng/g ww) in urine from female dam exposed via diet to 0.1, 1, or 6 mg/kg body weight of the MARBLES PCB mixture.

| **PCB/OH-PCB abbreviations** | **0.1 mg/kg (n=6)** | | | **1 mg/kg (n=5)** | | | **6 mg/kg (n=6)** | | |
| --- | --- | --- | --- | --- | --- | --- | --- | --- | --- |
|  | **Mean** | **SD** | **f** | **Mean** | **SD** | **f** | **Mean** | **SD** | **f** |
| PCB11 |  |  |  | 2.28 | 1.66 | 5 | 6.14 | 2.03 | 3 |
| 2-OH-PCB11 |  |  |  |  |  |  |  |  |  |
| PCB28 | 17.8 | 7.7 | 3 | 115 | 74 | 4 | 250 | 199 | 6 |
| 6-OH-PCB11 |  |  |  | 0.97 | 0.20 | 2 | 1.05 | 0.07 | 3 |
| PCB52 |  |  |  | 0.29 |  | 1 |  |  |  |
| 2'-OH-PCB28 | 0.07 | 0.03 | 6 | 0.44 | 0.24 | 5 | 1.00 | 0.59 | 6 |
| 5-OH-PCB11 |  |  |  | 0.44 |  | 1 | 0.26 | 0.18 | 2 |
| 5,6-OH-PCB11 |  |  |  |  |  |  |  |  |  |
| 2,5-OH-PCB11 |  |  |  |  |  |  |  |  |  |
| 4-OH-PCB11 | 9.50 | 7.44 | 4 | 40.0 | 24.4 | 5 | 56.7 | 31.7 | 5 |
| 3-OH-PCB28 | 0.66 | 0.11 | 3 | 6.30 | 2.84 | 5 | 17.3 | 10.3 | 6 |
| PCB95 |  |  |  |  |  |  |  |  |  |
| PCB84 |  |  |  |  |  |  |  |  |  |
| 3'-OH-PCB28 | 8.29 | 1.74 | 3 | 93.3 | 52.0 | 5 | 176 | 134 | 6 |
| 5-OH-PCB28 | 8.94 | 3.00 | 3 | 76.5 | 44.3 | 5 | 146 | 82 | 6 |
| 4'-OH-PCB25 |  |  |  |  |  |  |  |  |  |
| PCB101 |  |  |  |  |  |  | 1.34 |  | 2 |
| 4,5-OH-PCB11 |  |  |  |  |  |  |  |  |  |
| 3-OH-PCB103 |  |  |  |  |  |  |  |  |  |
| 4-OH-PCB52 |  |  |  | 0.40 | 0.18 | 5 | 0.86 | 0.52 | 6 |
| 6'-OH-PCB101 |  |  |  |  |  |  |  |  |  |
| PCB135 |  |  |  |  |  |  |  |  |  |
| PCB149 |  |  |  |  |  |  |  |  |  |
| 5-OH-PCB95 |  |  |  |  |  |  |  |  |  |
| PCB118 |  |  |  | 3.82 | 1.35 | 2 | 6.22 | 4.12 | 5 |
| 4'-OH-PCB95 |  |  |  |  |  |  |  |  |  |
| 4-OH-PCB95 |  |  |  |  |  |  |  |  |  |
| 4,5-OH-PCB95 |  |  |  |  |  |  | 0.06 | 0.04 | 4 |
| PCB153 |  |  |  |  |  |  | 3.04 |  | 2 |
| PCB138 |  |  |  | 1.60 | 0.82 | 2 | 2.47 | 1.47 | 4 |
| 4'-OH-PCB101 |  |  |  |  |  |  |  |  |  |
| 4,4'-OH-PCB52 |  |  |  |  |  |  |  |  |  |
| 3-OH-PCB118 | 0.16 | 0.04 | 6 |  |  |  |  |  |  |
| 3-OH-PCB153 |  |  |  |  |  |  |  |  |  |
| 4-OH-PCB146 |  |  |  |  |  |  |  |  |  |
| 3'-OH-PCB138 |  |  |  |  |  |  |  |  |  |
| PCB180 | 0.18 |  | 1 | 0.92 | 0.90 | 5 | 1.92 | 1.47 | 6 |
| 5-OH-PCB138 |  |  |  | 0.03 | 0.01 | 4 | 0.06 | 0.05 | 6 |
| 3'-OH-PCB180 |  |  |  |  |  |  |  |  |  |
| **Total PCB** | 18.0 |  |  | 124 |  |  | 271 |  |  |
| **Total OH-PCB** | 27.6 |  |  | 218 |  |  | 400 |  |  |
| **Total** | 45.6 |  |  | 342 |  |  | 671 |  |  |

f: detection frequency. Empty boxes indicate values below LOD (limit of detection) or not available.

**Table S11**. The levels of PCB and OH-PCB (ng/g ww tissue) in adipose tissue from female dam exposed via diet to 0.1, 1, or 6 mg/kg body weight of the MARBLES PCB mixture.

| **PCB/OH-PCB abbreviations** | **0.1 mg/kg (n=6)** | | | **1 mg/kg (n=5)** | | | **6 mg/kg (n=5)** | | |
| --- | --- | --- | --- | --- | --- | --- | --- | --- | --- |
|  | **Mean** | **SD** | **f** | **Mean** | **SD** | **f** | **Mean** | **SD** | **f** |
| PCB11 | 13.3 | 5.1 | 4 | 48.0 | 58.7 | 4 | 160 | 117 | 4 |
| 2-OH-PCB11 | 0.01 |  | 1 |  |  |  | 0.01 | 0.00 | 2 |
| PCB28 | 847 | 624 | 6 | 5040 | 2670 | 5 | 12000 | 6110 | 5 |
| 6-OH-PCB11 | 0.00 |  | 1 |  |  |  | 0.00 |  | 1 |
| PCB52 | 55.9 | 45.5 | 6 | 378 | 209 | 5 | 1100 | 648 | 5 |
| 2'-OH-PCB28 |  |  |  | 0.06 | 0.02 | 4 | 0.12 | 0.05 | 5 |
| 5-OH-PCB11 | 0.0005 |  | 1 |  |  |  | 0.0005 | 0.0001 | 2 |
| 5,6-OH-PCB11 | 0.0004 |  | 1 | 0.02 | 0.00 | 4 | 0.0005 | 0.0001 | 2 |
| 2,5-OH-PCB11 | 0.0004 |  | 1 | 0.02 | 0.01 | 4 | 0.0005 | 0.0001 | 2 |
| 4-OH-PCB11 | 0.0005 |  | 1 | 0.42 | 0.20 | 4 | 3.67 | 2.20 | 3 |
| 3-OH-PCB28 | 0.21 | 0.16 | 4 | 1.38 | 1.34 | 5 | 3.26 | 1.73 | 5 |
| PCB95 | 6.84 | 5.75 | 6 | 46.7 | 33.2 | 5 | 171 | 99 | 5 |
| PCB84 | 0.52 | 0.50 | 6 | 2.62 | 1.74 | 5 | 12.5 | 8.3 | 5 |
| 3'-OH-PCB28 | 0.58 | 0.50 | 6 | 1.62 | 0.51 | 5 | 6.79 | 4.04 | 5 |
| 5-OH-PCB28 | 0.34 | 0.31 | 6 | 0.57 | 0.16 | 5 | 2.74 | 1.81 | 5 |
| 4'-OH-PCB25 | 0.0007 |  | 1 |  |  |  | 0.0007 | 0.0001 | 2 |
| PCB101 | 35.4 | 25.8 | 6 | 311 | 215 | 5 | 1010 | 586 | 5 |
| 4,5-OH-PCB11 |  |  |  |  |  |  |  |  |  |
| 3-OH-PCB103 |  |  |  |  |  |  | 0.04 | 0.02 | 4 |
| 4-OH-PCB52 | 0.42 | 0.20 | 6 | 1.47 | 0.50 | 5 | 4.65 | 2.76 | 5 |
| 6'-OH-PCB101 |  |  |  |  |  |  |  |  |  |
| PCB135 | 2.65 | 1.55 | 6 | 17.4 | 10.7 | 5 | 61.8 | 34.9 | 5 |
| PCB149 | 6.46 | 3.88 | 6 | 40.5 | 26.9 | 5 | 139 | 69 | 5 |
| 5-OH-PCB95 |  |  |  |  |  |  | 0.22 | 0.12 | 3 |
| PCB118 | 147 | 84 | 6 | 1810 | 1550 | 5 | 4880 | 3740 | 5 |
| 4'-OH-PCB95 |  |  |  |  |  |  | 0.17 | 0.11 | 3 |
| 4-OH-PCB95 |  |  |  |  |  |  | 0.51 | 0.18 | 5 |
| 4,5-OH-PCB95 |  |  |  | 0.09 | 0.06 | 2 | 0.09 | 0.02 | 3 |
| PCB153 | 87.1 | 31.9 | 6 | 1100 | 953 | 5 | 2710 | 1650 | 5 |
| PCB138 | 35.6 | 14.9 | 6 | 486 | 452 | 5 | 1250 | 968 | 5 |
| 4'-OH-PCB101 |  |  |  | 0.15 | 0.05 | 5 | 0.52 | 0.28 | 5 |
| 4,4'-OH-PCB52 | 0.0007 |  | 1 |  |  |  | 0.0007 | 0.0001 | 2 |
| 3-OH-PCB118 | 1.09 |  | 1 | 0.76 | 0.51 | 5 | 2.31 | 1.03 | 5 |
| 3-OH-PCB153 | 0.26 | 0.48 | 6 | 0.42 | 0.21 | 5 | 1.46 | 1.04 | 5 |
| 4-OH-PCB146 |  |  |  | 0.06 | 0.02 | 5 |  |  |  |
| 3'-OH-PCB138 | 0.28 |  | 1 | 0.12 | 0.06 | 5 | 0.33 | 0.29 | 5 |
| PCB180 | 125 | 51 | 6 | 1440 | 1000 | 5 | 4240 | 2020 | 5 |
| 5-OH-PCB138 |  |  |  |  |  |  |  |  |  |
| 3'-OH-PCB180 | 0.22 |  | 1 | 0.09 | 0.05 | 5 | 0.23 | 0.13 | 5 |
| **Total PCB** | 1363 |  |  | 10700 |  |  | 27700 |  |  |
| **Total OH-PCB** | 3.41 |  |  | 7.25 |  |  | 27.1 |  |  |
| **Total** | 1370 |  |  | 10700 |  |  | 27700 |  |  |

f: detection frequency. Empty boxes indicate values below LOD (limit of detection) or not available.

**Table S12**. The levels of PCB and OH-PCB (ng/g ww) in blood from female dam exposed via diet to 0.1, 1, or 6 mg/kg body weight of the MARBLES PCB mixture.

| **PCB/OH-PCB abbreviations** | **0.1 mg/kg (n=6)** | | | **1 mg/kg (n=5)** | | | **6 mg/kg (n=5)** | | |
| --- | --- | --- | --- | --- | --- | --- | --- | --- | --- |
|  | **Mean** | **SD** | **f** | **Mean** | **SD** | **f** | **Mean** | **SD** | **f** |
| PCB11 | 0.64 |  | 1 | 30.9 | 37.5 | 2 | 4.97 | 3.46 | 3 |
| 2-OH-PCB11 |  |  |  |  |  |  |  |  |  |
| PCB28 | 3.95 | 2.53 | 6 | 50.0 | 65.9 | 5 | 158 | 108 | 5 |
| 6-OH-PCB11 |  |  |  |  |  |  | 0.07 | 0.05 | 4 |
| PCB52 | 0.45 | 0.18 | 4 | 4.42 | 5.57 | 5 | 12.9 | 8.5 | 5 |
| 2'-OH-PCB28 |  |  |  | 0.10 | 0.05 | 4 | 0.28 | 0.15 | 5 |
| 5-OH-PCB11 |  |  |  |  |  |  | 1.54 | 1.41 | 3 |
| 5,6-OH-PCB11 |  |  |  | 0.01 | 0.00 | 4 |  |  |  |
| 2,5-OH-PCB11 |  |  |  | 0.01 | 0.00 | 5 |  |  |  |
| 4-OH-PCB11 | 0.46 | 0.42 | 5 | 0.99 | 0.72 | 4 | 22.2 | 25.3 | 3 |
| 3-OH-PCB28 | 0.61 | 0.27 | 3 | 1.12 | 0.29 | 5 | 5.48 | 2.97 | 5 |
| PCB95 |  |  |  | 1.45 | 1.81 | 3 | 2.04 | 1.46 | 4 |
| PCB84 | 0.05 |  | 1 | 1.76 |  | 1 | 0.16 | 0.13 | 3 |
| 3'-OH-PCB28 | 2.36 | 3.42 | 6 | 13.9 | 16.4 | 5 | 60.3 | 58.4 | 5 |
| 5-OH-PCB28 | 1.96 | 3.54 | 6 | 10.3 | 16.6 | 5 | 39.8 | 34.1 | 5 |
| 4'-OH-PCB25 |  |  |  | 0.30 | 0.11 | 3 |  |  |  |
| PCB101 | 0.39 | 0.01 | 2 | 2.88 | 4.46 | 5 | 6.96 | 7.12 | 5 |
| 4,5-OH-PCB11 |  |  |  |  |  |  |  |  |  |
| 3-OH-PCB103 |  |  |  | 0.02 | 0.01 | 4 | 0.05 | 0.03 | 5 |
| 4-OH-PCB52 | 4.69 | 1.20 | 2 | 12.3 | 4.2 | 5 | 42.8 | 19.2 | 5 |
| 6'-OH-PCB101 |  |  |  |  |  |  |  |  |  |
| PCB135 | 0.04 | 0.03 | 5 | 0.92 | 1.85 | 5 | 0.58 | 0.75 | 5 |
| PCB149 |  |  |  | 4.19 | 4.64 | 2 | 3.27 | 2.19 | 3 |
| 5-OH-PCB95 |  |  |  |  |  |  | 1.17 | 1.30 | 3 |
| PCB118 | 0.66 | 0.30 | 5 | 4.86 | 4.85 | 5 | 20.9 | 15.8 | 5 |
| 4'-OH-PCB95 |  |  |  | 0.05 | 0.03 | 5 | 0.42 | 0.41 | 4 |
| 4-OH-PCB95 |  |  |  | 1.32 | 0.53 | 5 | 5.06 | 2.95 | 5 |
| 4,5-OH-PCB95 |  |  |  | 0.09 | 0.03 | 5 | 0.33 | 0.18 | 5 |
| PCB153 | 1.29 | 0.20 | 3 | 5.84 | 5.34 | 5 | 16.0 | 12.5 | 5 |
| PCB138 | 0.85 | 0.35 | 5 | 3.66 | 2.76 | 5 | 8.78 | 6.38 | 5 |
| 4'-OH-PCB101 | 0.14 | 0.09 | 6 | 0.64 | 0.12 | 5 | 2.38 | 1.04 | 5 |
| 4,4'-OH-PCB52 |  |  |  | 0.15 | 0.04 | 5 | 0.25 | 0.11 | 4 |
| 3-OH-PCB118 | 0.22 | 0.30 | 6 | 0.70 | 0.30 | 5 | 3.29 | 1.24 | 5 |
| 3-OH-PCB153 | 0.41 | 0.66 | 5 | 0.60 | 0.17 | 5 | 2.35 | 1.27 | 5 |
| 4-OH-PCB146 |  |  |  | 0.12 |  | 1 | 0.15 | 0.02 | 4 |
| 3'-OH-PCB138 | 0.04 | 0.06 | 6 | 0.09 | 0.05 | 5 | 0.33 | 0.20 | 5 |
| PCB180 | 1.55 | 0.88 | 6 | 6.78 | 4.61 | 5 | 22.6 | 15.8 | 5 |
| 5-OH-PCB138 |  |  |  |  |  |  | 0.05 | 0.03 | 5 |
| 3'-OH-PCB180 | 0.18 | 0.25 | 3 | 0.15 | 0.10 | 5 | 0.51 | 0.27 | 5 |
| **Total PCB** | 9.88 |  |  | 118 |  |  | 257 |  |  |
| **Total OH-PCB** | 11.1 |  |  | 43 |  |  | 189 |  |  |
| **Total** | 20.9 |  |  | 161 |  |  | 446 |  |  |

f: detection frequency. Empty boxes indicate values below LOD (limit of detection) or not available.

**Table S13**. The levels of PCB and OH-PCB (ng/g ww tissue) in the liver from female dam exposed via diet to 0.1, 1, or 6 mg/kg body weight of the MARBLES PCB mixture.

| **PCB/OH-PCB abbreviations** | **0.1 mg/kg (n=6)** | | | **1 mg/kg (n=5)** | | | **6 mg/kg (n=5)** | | |
| --- | --- | --- | --- | --- | --- | --- | --- | --- | --- |
|  | **Mean** | **SD** | **f** | **Mean** | **SD** | **f** | **Mean** | **SD** | **f** |
| PCB11 | 0.78 | 0.29 | 4 | 9.88 | 13.32 | 3 | 14.0 | 9.5 | 4 |
| 2-OH-PCB11 | 0.002 |  | 1 | 0.002 | 0.0002 | 4 | 0.003 | 0.0003 | 3 |
| PCB28 | 96.2 | 69.0 | 6 | 495 | 225 | 5 | 1790 | 856 | 5 |
| 6-OH-PCB11 | 0.0002 |  | 1 | 0.0002 | 0.00 | 3 | 0.0003 | 0.00 | 3 |
| PCB52 | 2.83 | 2.38 | 6 | 13.6 | 6.6 | 5 | 69.8 | 38.0 | 5 |
| 2'-OH-PCB28 | 0.01 | 0.01 | 6 | 0.08 | 0.03 | 5 | 0.20 | 0.09 | 5 |
| 5-OH-PCB11 | 0.0002 |  | 1 | 0.0002 | 0.00 | 4 | 0.35 | 0.41 | 5 |
| 5,6-OH-PCB11 | 0.0001 |  | 1 | 0.0002 | 0.00 | 5 | 0.0002 | 0.00 | 3 |
| 2,5-OH-PCB11 | 0.0002 |  | 1 | 0.0002 | 0.00 | 5 | 0.0002 | 0.00 | 3 |
| 4-OH-PCB11 | 0.63 | 0.42 | 4 | 2.98 | 3.62 | 4 | 12.5 | 12.3 | 5 |
| 3-OH-PCB28 | 0.62 | 0.60 | 6 | 7.32 | 3.30 | 5 | 18.5 | 8.6 | 5 |
| PCB95 | 1.03 | 0.37 | 4 | 1.85 | 0.82 | 5 | 11.4 | 6.3 | 5 |
| PCB84 | 0.09 | 0.01 | 2 | 0.15 | 0.04 | 2 | 0.60 | 0.42 | 5 |
| 3'-OH-PCB28 | 14.5 | 17.0 | 6 | 145 | 28 | 5 | 318 | 161 | 5 |
| 5-OH-PCB28 | 9.51 | 10.99 | 5 | 60.2 | 13.0 | 5 | 141 | 74 | 5 |
| 4'-OH-PCB25 | 0.0002 |  | 1 |  |  |  | 0.46 | 0.31 | 5 |
| PCB101 | 3.31 | 2.69 | 6 | 11.0 | 4.4 | 5 | 77.2 | 46.4 | 5 |
| 4,5-OH-PCB11 | 0.06 | 0.08 | 2 | 0.0002 | 0.00 | 5 | 0.0003 | 0.00 | 3 |
| 3-OH-PCB103 |  |  |  | 0.03 | 0.01 | 5 | 0.07 | 0.04 | 5 |
| 4-OH-PCB52 | 0.54 | 0.47 | 6 | 4.23 | 1.93 | 5 | 14.2 | 9.1 | 5 |
| 6'-OH-PCB101 |  |  |  |  |  |  |  |  |  |
| PCB135 | 0.32 | 0.09 | 4 | 0.60 | 0.31 | 5 | 5.95 | 5.15 | 5 |
| PCB149 | 0.79 | 0.26 | 4 | 1.49 | 0.66 | 5 | 13.8 | 10.2 | 5 |
| 5-OH-PCB95 |  |  |  | 0.22 | 0.15 | 4 | 1.11 | 1.11 | 5 |
| PCB118 | 11.3 | 7.4 | 6 | 79.7 | 46.3 | 5 | 379 | 157 | 5 |
| 4'-OH-PCB95 |  |  |  | 0.16 | 0.09 | 5 | 1.33 | 1.28 | 5 |
| 4-OH-PCB95 |  |  |  | 0.35 | 0.16 | 5 | 1.10 | 0.56 | 5 |
| 4,5-OH-PCB95 |  |  |  | 0.14 | 0.03 | 5 | 0.58 | 0.43 | 5 |
| PCB153 | 9.62 | 5.81 | 6 | 49.8 | 20.5 | 5 | 287 | 150 | 5 |
| PCB138 | 23.8 | 19.1 | 6 | 79.1 | 37.7 | 5 | 212 | 96 | 5 |
| 4'-OH-PCB101 |  |  |  | 0.58 | 0.09 | 5 | 3.08 | 1.48 | 5 |
| 4,4'-OH-PCB52 | 0.0001 |  | 1 | 0.0002 | 0.00 | 5 | 0.0002 | 0.0001 | 4 |
| 3-OH-PCB118 | 0.93 | 1.19 | 6 | 3.51 | 1.03 | 5 | 11.6 | 4.3 | 5 |
| 3-OH-PCB153 | 1.36 | 2.00 | 3 | 0.68 | 0.27 | 5 | 2.68 | 1.68 | 5 |
| 4-OH-PCB146 |  |  |  |  |  |  |  |  |  |
| 3'-OH-PCB138 | 0.27 | 0.57 | 6 | 0.47 | 0.20 | 5 | 1.71 | 1.23 | 5 |
| PCB180 | 52.3 | 36.1 | 6 | 199 | 78 | 5 | 521 | 190 | 5 |
| 5-OH-PCB138 |  |  |  | 0.03 | 0.01 | 5 | 0.08 | 0.05 | 4 |
| 3'-OH-PCB180 | 0.46 |  | 1 | 0.19 | 0.07 | 5 | 0.58 | 0.35 | 5 |
| **Total PCB** | 202 |  |  | 941 |  |  | 3380 |  |  |
| **Total OH-PCB** | 29 |  |  | 226 |  |  | 529 |  |  |
| **Total** | 231 |  |  | 1170 |  |  | 3910 |  |  |

f: detection frequency. Empty boxes indicate values below LOD (limit of detection) or not available.

**References**

Alam, S., Carter, G., Krager, K., Li, X., Lehmler, H.-J. and Aykin-Burns, N. (2018). PCB11 metabolite, 3,3’-dichlorobiphenyl-4-ol, exposure alters the expression of genes governing fatty acid metabolism in the absence of functional sirtuin 3: examining the contribution of MnSOD. *Antioxidants* 7, E121, <https://doi.org/10.3390/antiox7090121>.

Black, T.H. (1983). The preparation and reactions of diazomethane. *Aldrichim Acta* 16, 3–10.

Dhakal, K., He, X., Lehmler, H.J., Teesch, L.M., Duffel, M.W. and Robertson, L.W. (2012). Identification of sulfated metabolites of 4-chlorobiphenyl (PCB3) in the serum and urine of male rats. *Chem Res Toxicol* 25, 2796–2804, <https://doi.org/10.1021/tx300416v>.

Dhakal, R., Li, X., Parkin, S.R. and Lehmler, H.-J. (2020). Synthesis of mono-and dimethoxylated polychlorinated biphenyls derivatives starting from fluoroarene derivatives. *Environ Sci Pollut Res* 27, 8905–8925, <https://doi.org/10.1007/s11356-019-07133-3>.

Joshi, S.N., Vyas, S.M., Duffel, M.W., Parkin, S. and Lehmler, H.-J. (2011). Synthesis of sterically hindered polychlorinated biphenyl derivatives. *Synthesis*, 1045–1054, <https://doi.org/10.1055/s-0030-1258454>.

Kania-Korwel, I., Zhao, H., Norstrom, K., Li, X., Hornbuckle, K.C. and Lehmler, H.-J. (2008). Simultaneous extraction and clean-up of PCBs and their metabolites from small tissue samples using pressurized liquid extraction. *J Chromatogr A* 1214, 37–46, <https://doi.org/10.1016/j.chroma.2008.10.089>.

Lehmler, H.J. and Robertson, L.W. (2001a). Synthesis of hydroxylated PCB metabolites with the Suzuki-coupling. *Chemosphere* 45, 1119–1127.

Lehmler, H.J. and Robertson, L.W. (2001b). Synthesis of polychlorinated biphenyls (PCBs) using the Suzuki-coupling. *Chemosphere* 45, 137–143.

Li, X., Holland, E.B., Feng, W., Zheng, J., Dong, Y., Pessah, I.N., Duffel, M.W., Robertson, L.W. and Lehmler, H.-J. (2018). Authentication of synthetic environmental contaminants and their (bio)transformation products in toxicology: polychlorinated biphenyls as an example. *Environ Sci Pollut Res* 25, 16508–16521, <https://doi.org/10.1007/s11356-017-1162-0>.

Li, X., Parkin, S., Duffel, M.W., Robertson, L.W. and Lehmler, H.-J. (2010). An efficient approach to sulfate metabolites of polychlorinated biphenyls. *Environ Int* 36, 843–848, <https://doi.org/10.1016/j.envint.2009.02.005>.

Marek, R.F., Thorne, P.S., Wang, K., Dewall, J. and Hornbuckle, K.C. (2013). PCBs and OH-PCBs in serum from children and mothers in urban and rural U.S. communities. *Environ Sci Technol* 47, 3353–3361, <https://doi.org/10.1021/es304455k>.

McLean, M.R., Bauer, U., Amaro, A.R. and Robertson, L.W. (1996). Identification of catechol and hydroquinone metabolites of 4-monochlorobiphenyl. *Chem Res Toxicol* 9, 158–164, <https://doi.org/10.1021/tx950083a>.

Rodriguez, E.A., Li, X., Lehmler, H.J., Robertson, L.W. and Duffel, M.W. (2016). Sulfation of lower chlorinated polychlorinated biphenyls increases their affinity for the major drug-binding sites of human serum albumin. *Environ Sci Technol* 50, 5320–5327, <https://doi.org/10.1021/acs.est.6b00484>.

Saktrakulkla, P., Dhakal, R.C., Lehmler, H.J. and Hornbuckle, K.C. (2020). A semi-target analytical method for quantification of OH-PCBs in environmental samples. *Environ Sci Pollut Res* 27, 8859–8871, <https://doi.org/10.1007/s11356-019-05775-x>.

Wang, H., Bullert, A.J., Li, X., Stevens, H., Klingelhutz, A.J., Ankrum, J.A., Adamcakova-Dodd, A., Thorne, P.S. and Lehmler, H.J. (2023). Use of a polymeric implant system to assess the neurotoxicity of subacute exposure to 2,2',5,5'-tetrachlorobiphenyl-4-ol, a human metabolite of PCB 52, in male adolescent rats. *Toxicology* 500, 153677, <https://doi.org/10.1016/j.tox.2023.153677>.

Zhai, G.S., Lehmler, H.J. and Schnoor, J.L. (2011). New hydroxylated metabolites of 4-monochlorobiphenyl in whole poplar plants. *Chem Cent J* 5, <https://doi.org/10.1186/1752-153X-5-87>.
